# Validating the Effectiveness of Forest Therapy Programs for Middle-Aged Korean Women: A Systematic Review and Meta-Analytic Approach

**DOI:** 10.3390/healthcare14111569

**Published:** 2026-06-03

**Authors:** Young-Ho Lee, Gyeong-Min Min, Pyeong-Sik Yeon

**Affiliations:** 1 Graduate Department of Forest Therapy, Chungbuk National University, Cheongju 28644, Republic of Korea; yhleec@chungbuk.ac.kr (Y.-H.L.); akwjs5019@chungbuk.ac.kr (G.-M.M.); 2 Department of Forest Sciences, Chungbuk National University, Cheongju 28644, Republic of Korea

**Keywords:** forest therapy, meta-analysis, middle-aged Korean women, nature-based intervention, health promotion

## Abstract

**Background/Objectives:** Middle-aged Korean women (aged 40–65 years) face compounded physiological and psychosocial health burdens, yet controlled evidence for non-pharmacological interventions in this population remains limited. This systematic review and meta-analysis aimed to quantify the effects of forest therapy on health-related outcomes in middle-aged Korean women and to identify program characteristics associated with differential therapeutic effects. **Methods:** Ten databases were searched for controlled studies published from January 2000 to February 2025 following PRISMA 2020 and PICOTS-SD criteria; only controlled studies conducted in Korea were included in the meta-analysis. Of 9563 records screened, 24 controlled Korean studies (RCT, *n* = 13; NRCT, *n* = 11; k = 128 effect sizes) met inclusion criteria. A three-level random-effects model with robust variance estimation (RVE) was used as the primary analysis. **Results:** The primary three-level RVE model, applied to 24 controlled Korean studies, yielded a pooled Hedges’ g = 0.596 (95% CI: 0.432–0.760); a supplementary standard random-effects model yielded g = 0.542 (95% CI: 0.420–0.664). Substantial heterogeneity and potential publication bias were observed; overall evidence certainty was rated Low (GRADE). **Conclusions:** These findings provide preliminary, low-certainty evidence (overall GRADE: Low) that forest therapy may benefit middle-aged Korean women. They do not justify broad clinical or policy adoption at present. High-quality, independently conducted international RCTs and standardized trials outside Korea are required to confirm and generalize these findings.

## 1. Introduction

Life expectancy in the Republic of Korea has been continuously increasing, driven by dramatic advances in healthcare technology and improved living standards. As of 2022, the life expectancy for Korean women had reached 85.6 years [1]. Middle-aged women (aged 40–65 years) are now recognized as a critical demographic that determines individual quality of life and socioeconomic outcomes. The World Health Organization (WHO) emphasizes that unhealthy aging and gender-based health inequalities impose significant economic burdens owing to productivity losses and rising healthcare expenditures [2]. In 2024, middle-aged Korean women constituted approximately 20.1% of the total population of the Republic of Korea [3]. This group experiences simultaneous physiological changes, including decreased estrogen, vasomotor symptoms, insomnia, and decreased bone density, along with psychosocial transitions, such as “empty-nest syndrome,” reduced occupational roles, and the burden of caring for older adult parents [4].

Korean women are reported to experience a decline in life satisfaction from 63.9% in their 50s to 51.0% in their 60s [5]. Furthermore, the 12-month prevalence of major depressive disorder among middle-aged Korean women is 1.8 to 3.3 times higher than that among age-matched men [6]. This pattern is consistent with global concerns regarding women in transitional life stages, including the climacteric period, who may benefit from non-pharmacological health interventions [7].

Health issues among middle-aged women, including menopausal symptoms and psychological distress, impose a significant socioeconomic burden on the public health system [2,4]. Although pharmacological treatments are commonly used, there is a growing need for non-pharmacological nature-based interventions that offer high accessibility and minimal side effects.

Nature-based interventions have attracted increasing attention as potentially accessible and low-burden approaches to address these public health challenges [8,9]. Forest therapy, defined as a structured program utilizing therapeutic forest elements (e.g., landscape, sunlight, temperature, and humidity), is gaining attention as a safe and accessible non-pharmacological intervention. South Korea has developed an advanced domestic infrastructure for forest therapy under the Forest Culture and Recreation Act (2010), including “Healing Forests” and a national certification system for Forest Therapy Instructors [10]. The observed effects may be interpreted in light of stress recovery theory, attention restoration theory, and the biophilia hypothesis: Ulrich’s Stress Recovery Theory (SRT), Kaplan and Kaplan’s Attention Restoration Theory (ART), and the Biophilia Hypothesis [11,12,13].

Although prior meta-analyses have focused on general adult populations or specific clinical outcomes [8,14,15,16], systematic evidence from environmental health and forestry research has consistently demonstrated that forest immersion significantly improves physical and mental health markers across diverse populations [17]. Furthermore, structured prescription models for forest therapy that incorporate certified instructors, standardized program elements, and population-specific dosages have been proposed as a framework to maximize therapeutic outcomes in clinical and preventive settings [18]. Nevertheless, few studies have designated middle-aged Korean women as an independent subject group or elucidated the biopsychosocial mechanisms unique to this demographic. Although the initial scope of this review encompassed middle-aged women worldwide, the systematic search identified 36 studies that met the systematic review criteria (34 domestic and 2 international). However, both international studies were excluded from the meta-analysis because they lacked a comparison group, which is a mandatory requirement for controlled-study meta-analysis. After excluding domestic research with single-group pre–post designs, 24 controlled studies were included in the meta-analysis, all of which were Korean domestic publications. This outcome is attributable to the study design filter applied at the meta-analysis stage rather than to any deliberate geographic restriction. Thus, it reflects that Korea has developed a legislatively supported and nationally standardized healing-forest infrastructure, which has enabled the accumulation of a relatively large body of controlled evidence for this population. The lack of international controlled studies limits the generalizability of the findings and indicates that additional research in other countries is needed. Therefore, this study provides a rigorous evidence base for forest therapy in the Korean healing-forest context and establishes a methodological template for future international replication studies. In light of these evidence gaps and Korea’s unique healing-forest infrastructure, the present study was designed to provide the first population-specific quantitative synthesis of forest therapy for middle-aged women. Specifically, the objectives of this study were as follows: (1) to quantify the pooled effect of forest therapy on health-related outcomes (psychological, physiological, and physical) in middle-aged Korean women using a meta-analytic approach; (2) to examine whether intervention type, program format, setting, and dosage characteristics moderate the pooled effect size within the Korean evidence base; and (3) to assess the certainty of evidence using the GRADE framework and identify priority directions for future high-quality research, including international replication.

## 2. Materials and Methods

This systematic review and meta-analysis was conducted in strict accordance with the Preferred Reporting Items for Systematic Reviews and Meta-Analyses (PRISMA) 2020 guidelines. The completed PRISMA 2020 checklist is provided in Appendix A (Table A3).

Protocol Registration: This review was registered in INPLASY (International Platform of Registered Systematic Review and Meta-analysis Protocols; registration number: INPLASY202650156; registration date: 28 May 2026). A protocol summary documenting the pre-specified analytical framework is provided in the Supplementary Materials (Document S1). The primary eligibility criteria (PICOTS-SD framework), database selection, search terms, and key statistical analysis approaches (DerSimonian–Laird random-effects model, Hedges’ *g*, 14 categorical moderator variables) were rigorously determined prior to the formal data extraction phase, thereby minimizing the risk of post hoc analytical decisions. Specifically, the PICOTS-SD criteria (Table 1) were finalized before search initiation on 1 March 2025; the decision to employ R 4.4.3 software utilizing the metafor 4.8-0 and robumeta 2.1 packages) for the primary three-level random-effects model with robust variance estimation (RVE), along with supplementary DL random-effects modeling via CMA v4.0 as a sensitivity check, was pre-specified; and no primary outcome or moderator variable was added following data extraction.

During manuscript preparation we used large language models (Claude 3.5 Sonnet; Gemini 1.5 Pro) solely for grammar and phrasing edits in the Discussion and for improving figure resolution. AI was not used for study selection, data extraction, statistical analyses, or substantive interpretation; all AI outputs were reviewed and edited by the authors.

### 2.1. Eligibility Criteria (PICOTS-SD)

Table 1 presents the PICOTS-SD eligibility criteria, which were prospectively defined before the literature search. Although the population (P) was initially defined broadly as middle-aged women (aged 40–65 years), the systematic search only identified 36 studies meeting the systematic review criteria, including 2 international studies. However, both international studies lacked a comparison group and were therefore excluded from the meta-analysis. Thus, all 24 studies included in the meta-analysis originated in Korea (see Section 3.1). Accordingly, the meta-analytic target population was limited to middle-aged Korean women, as the final controlled evidence base was derived entirely from Korean studies. Intervention (I) classified programs based on the operator, design purpose, and implementation setting of forest therapy interventions: first, field practice-centered programs planned by certified forest therapy instructors; second, research-designed programs with adjusted intervention intensity and content to meet study objectives; and third, indoor-based programs with spatial constraints as a variable. The comparator (C) comprised studies with a comparison group. The outcomes (O) were categorized as mental, physiological, and physical effects. The timeframe (T) was not restricted and was classified according to session count, session duration, residential (overnight) type, and same-day session type. The setting (S) was classified into general populations and disease or symptom groups. Finally, the study design (SD) included not only randomized controlled trials (RCTs) but also non-randomized controlled trials (NRCTs) and single-group pre–post experimental designs, considering the possibility of insufficient data in the systematic review process. Single-group pre–post designs were included in the systematic review but excluded from the meta-analysis. Studies targeting only urban parks or mixed genders without sex-stratified data were excluded.

### 2.2. Literature Search

**Figure 1:**
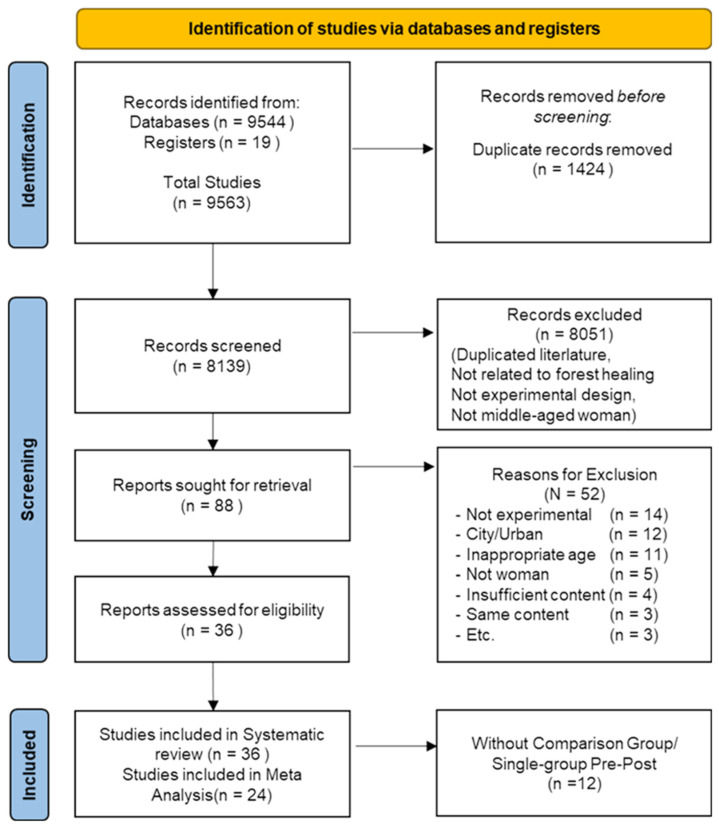
PRISMA Study Selection Flow Diagram.

A systematic search was conducted between 1 and 7 March 2025 (covering January 2000 to February 2025; languages restricted to Korean and English), in five domestic databases (RISS, DBpia, KISS, ScienceON, and National Assembly Library) and five international databases (Web of Science, Scopus, PubMed, MEDLINE, and EMBASE). Domestic search terms combined “forest” OR “forest experience” OR “forest activity” OR “forest play” OR “nature” OR “wood” OR “urban forest” OR “forest bathing” OR “forest therapy” AND “middle-aged women” OR “climacteric” OR “menopausal.” International search terms combined “forest” OR “forest therapy” OR “shinrin-yoku” AND “middle-aged wom*n” OR “menopausal” OR “climacteric.” Manual searches of the reference lists identified 19 additional records (total: 9563 records; Figure 1).

### 2.3. Study Selection and Data Extraction

Two researchers (Y.H.L and G.M.M.) independently reviewed all titles and abstracts, as well as full texts (*n* = 88). Inter-rater screening agreement was assessed using a 2 × 2 contingency table, yielding an observed agreement rate of 90.9% (both included: *n* = 32; both excluded: *n* = 48; disagreements: *n* = 8). Cohen’s κ was 0.812 (95% CI: 0.688–0.936), which indicated almost perfect agreement according to Landis and Koch’s [19] criteria. Disagreements (*n* = 8) were resolved by a third researcher (P.S.Y.) through structured consensus discussions. Data extraction items included bibliographic characteristics, participant demographics, intervention details, comparator types, outcome domains, and statistical parameters. Data were independently extracted by two researchers (Y.H.L. and G.M.M.). Any disagreements regarding extracted values were resolved through structured consensus discussions, and the study investigators did not directly seek any additional data.

### 2.4. Risk of Bias Assessment

Two independent researchers used RoB 2.0 (5 domains [20]) to assess the risk of bias in RCTs, with confirmed inter-rater reliability for bias judgments (*κ* > 0.70 for all domains). NRCTs were assessed using ROBINS-I (7 domains [21]). Considering the structural impossibility of blinding in experiential forest therapy, studies rated as “some concerns” in the blinding domain were included after sensitivity analyses confirmed that their inclusion or exclusion did not affect the overall conclusions.

### 2.5. Statistical Analysis

To comprehensively address the risk of intra-study statistical dependency arising from multiple outcomes per primary study, a three-level random-effects model (levels: sampling variance, effect-within-study, and between-study) fitted by REML with robust variance estimation (RVE, CR2 correction) was employed as our primary analytical framework. This advanced approach explicitly models the nested structure of the 128 effect sizes within the 24 primary studies without artificially inflating statistical precision. Consequently, for the 14 categorical moderator analyses (subgroup analyses), a standard random-effects model treating the 128 effect sizes as independent units was deliberately maintained strictly for exploratory purposes. This pragmatic decision was made to ensure model convergence and prevent critical degrees-of-freedom deficiencies (*df* < 4) within small-*k* subgroups, which frequently occur in three-level meta-regressions with numerous categorical predictors. The three-level random-effects model requires stable estimation of two variance components (within-study σ^2^_2_ and between-study σ^2^_3_); however, with *k* = 24 studies—some contributing as few as one to two effect sizes—the precision of the within-study variance component estimate may be limited. All subgroup findings should therefore be interpreted as exploratory and hypothesis-generating. To further ensure methodological robustness and comparability with prior literature, the independent-effects DerSimonian–Laird model was run as a secondary sensitivity check. Additional sensitivity analyses included a leave-one-out analysis at the individual effect level (*k* = 128) to evaluate the internal stability of the pooled overall estimate and ensure that no single data point disproportionately inverted the baseline therapeutic trend. Heterogeneity was quantified via *Tau*^2^ components and an approximate *I*^2^ decomposition (within- vs. between-study contributions), and 95% prediction intervals were computed. All analysis scripts and datasets are provided in the Supplementary Materials to ensure full reproducibility.

## 3. Results

### 3.1. Study Selection and Characteristics

Of 9563 records, 1424 duplicates were removed, 8051 were excluded after title/abstract screening, and 52 were excluded after full-text review (non-experimental, *n* = 14; urban/city only *n* = 12; inappropriate age *n* = 11; not female-only *n* = 5; insufficient data *n* = 4; same content *n* = 3; other *n* = 3). A total of 36 studies (34 domestic and 2 international) met the systematic review criteria (Figure 1). All 36 studies were included in the systematic review. However, two international studies were excluded from the meta-analysis because they did not include a comparison group, yielding only single-group pre–post data that did not meet the controlled study eligibility criterion for meta-analysis. After additionally excluding the remaining 10 domestic single-group pre–post designs, 24 controlled studies (RCT *n* = 13; NRCT *n* = 11; *k* = 128 effect sizes), all of which were Korean domestic publications, were meta-analyzed. Therefore, this outcome reflected the study design filter applied at the meta-analysis stage, specifically the requirement for a control group. The lack of international controlled evidence limits the broader generalizability of the findings and underscores the need for replication in diverse contexts.

The systematic review analyzed 36 domestic and international studies. RCTs accounted for the highest proportion (36%), whereas NRCTs and single-group pre–post designs accounted for 31% and 33%, respectively. Of the participants, 83% were general middle-aged Korean women without any specific diseases. Overall, 83% of the studies used same-day session formats, with residential (overnight) programs representing a minority (11%). The most common session duration was 1 h to less than 2 h (50%), weekly frequency was predominantly once per week (40%), and the most common program length fell within the 6-to-less-than-10-session range (34%, short-to-medium-term programs).

### 3.2. Risk of Bias

**Figure 2:**
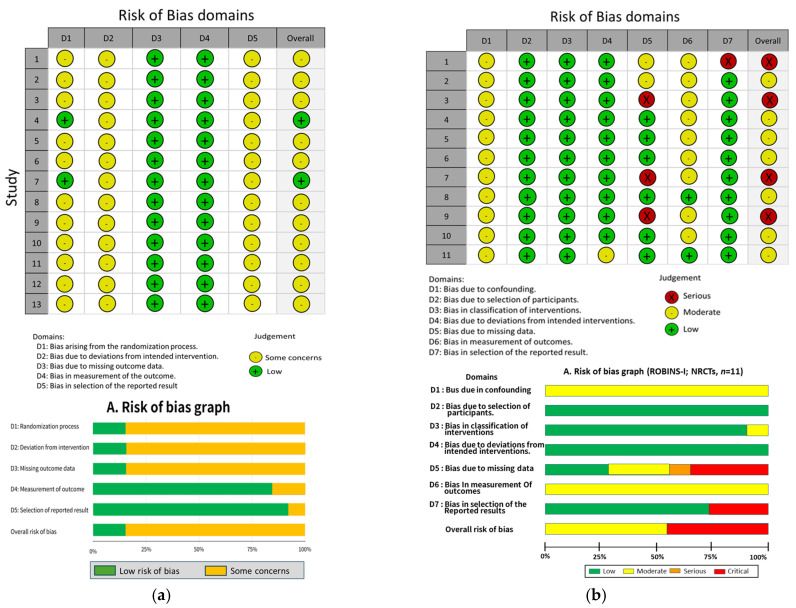
Risk of Bias Assessment Results. (**a**) RoB 2.0 for RCTs (*n* = 13); (**b**) ROBINS-I for NRCTs (*n* = 11).

A quality assessment of the meta-analysis was conducted on 24 studies (RCTs, *n* = 13; NRCTs, *n* = 11), excluding 12 studies without a comparison group (10 domestic single-group pre–post designs and 2 international studies). RCT quality was assessed using RoB 2.0, while NRCT quality was assessed using ROBINS-I (Figure 2). Among the assessed RCTs, 11 merely stated that randomization had occurred without describing the detailed implementation procedures. Consequently, allocation concealment was rated as “some concerns” in most studies. Additionally, the blinding domain was rated as ‘some concerns’ because the outcome assessors were aware of the intervention content. NRCTs have also presented serious blinding concerns because the researchers directly evaluated the outcomes. However, the practical nature of forest therapy programs inherently limits perfect physical blinding. Accordingly, all 24 studies were included in the final meta-analysis because no evidence of intentional result manipulation or selective reporting was identified.

### 3.3. Overall Effect Size, Heterogeneity, and Prediction Interval

To comprehensively address the risk of intra-study dependency, we employed a three-level random-effects model with robust variance estimation (RVE, CR2 correction) as our primary analytical framework. This advanced approach explicitly models the nested structure of multiple effect sizes within each primary study without artificially inflating the statistical precision.

Accounting for data dependency, the primary three-level RVE model revealed a robust overall effect size (Table 2) (SE = 0.081, 95% CI: 0.432–0.760, *p* < 0.001). According to Cohen’s *d* (or Hedges’ *g*) benchmark for effect size interpretation, an effect size of 0.2 is considered small, 0.5 is moderate, and 0.8 is large [22,23,24]. Therefore, the pooled estimate obtained in this study (*g* = 0.596, 95% CI: 0.432–0.760) indicates a statistically significant, moderate-to-large positive therapeutic effect of forest therapy programs on health outcomes in middle-aged Korean women. For sensitivity validation, the standard DL model yielded a highly consistent effect size of *g* = 0.542 (95% CI: 0.420–0.664), confirming that the therapeutic benefits remain stable regardless of the statistical framing. The convergence of the primary three-level RVE estimate (*g* = 0.596, 95% CI: 0.432–0.760) with the supplementary DerSimonian–Laird estimate (*g* = 0.542, 95% CI: 0.420–0.664) provides evidence of robustness across fundamentally different statistical assumptions regarding effect-size independence. Both models yielded statistically significant positive effects (*p* < 0.001), and their 95% confidence intervals overlapped substantially, indicating that the therapeutic signal is not an artifact of any single analytical framework. This conclusion was further corroborated by the leave-one-out analysis (*g* range: 0.462–0.678, all 24 permutations excluding zero), which confirmed that no single study disproportionately drove the pooled estimate. The 95% prediction interval (PI: −0.721 to 1.913) indicates substantial between-setting variability in true effects.

**Figure 3:**
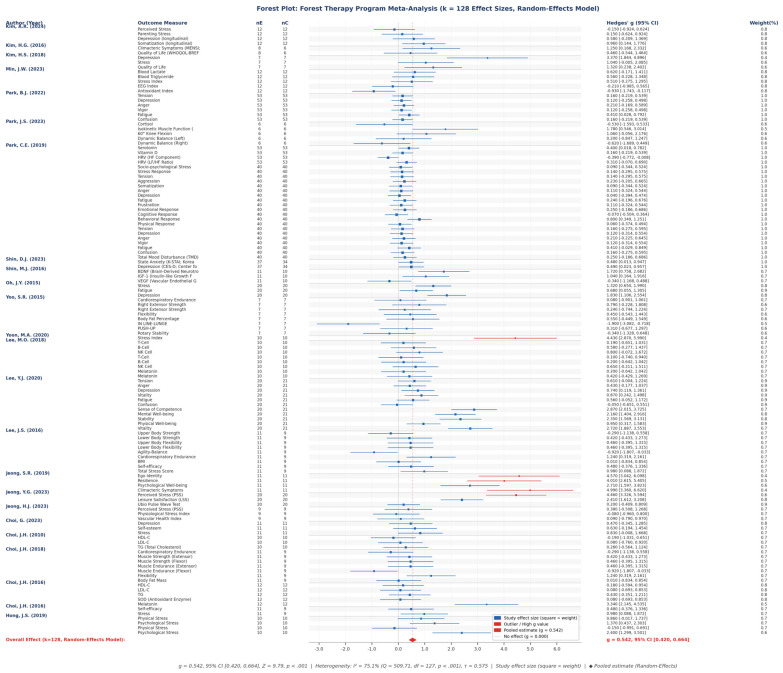
Forest Plot of the Included Studies. Note: The plot visually represents the supplementary standard random-effects model (*g* = 0.542, 95% CI: 0.420–0.664), which demonstrates high directional consistency with the primary three-level RVE model (*g* = 0.596, 95% CI: 0.432–0.760). Kim, A.R., 2024 [27], Kim, H.G., 2016 [28], Kim, H.S., 2018 [29], Min, J.W., 2023 [30], Park, B.J., 2022 [31], Park, J.S., 2023 [32], Park, C.E., 2019 [33], Shin, D.J., 2023 [34], Shin, M.J., 2016 [35], Oh, J.Y., 2015 [36], Yoo, S.R., 2015 [37], Yoon, M.A., 2020 [38], Lee, M.O., 2018 [39], Lee, Y.J., 2020 [40], Lee, J.S., 2016 [41], Jeong, S.R., 2019 [42], Jeong, Y.G., 2023 [43], Jeong, H.J., 2023 [44], Choi, G., 2023 [45], Choi, J.H., 2010 [46], Choi, J.H., 2018 [47], Choi, J.H., 2017 [48], Choi, J.H., 2016 [49], Hong, J.S., 2019 [50].

While the primary overall estimate was rigorously verified using the three-level RVE model, the visual Forest Plot (Figure 3) and baseline heterogeneity metrics (Q and *I*^2^) were deliberately retained under the traditional DerSimonian–Laird (DL) framework (Table 3). This hybrid approach was chosen to ensure direct comparability with prior forest therapy literature and to maximize graphical legibility for readers, given that the baseline magnitude of heterogeneity remained consistently severe across both analytical frameworks. Having confirmed the robustness of the primary pooled estimate through convergence of the three-level RVE and DerSimonian–Laird models, all subsequent subgroup (moderator) analyses reported in Section 3.6 were conducted using the standard random-effects model implemented in Comprehensive Meta-Analysis (CMA v4.0), as pre-specified, to ensure model convergence and prevent degrees-of-freedom deficiencies in small-*k* subgroups.

### 3.4. Publication Bias

**Figure 4:**
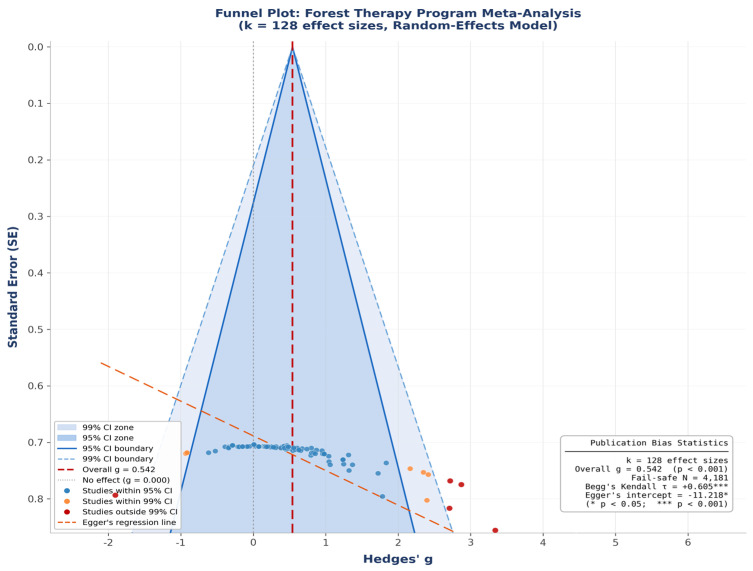
Funnel Plot.

Egger’s and Begg’s tests indicated funnel plot (Figure 4) asymmetry (τ = 0.18, *p* < 0.05; t = 3.42, *p* < 0.05). Trim-and-fill (Table 4) performs poorly under high heterogeneity [25,26] and estimated no imputed studies; thus such diagnostics are interpreted cautiously. Rosenthal’s Fail-safe N is large (11,511) but does not fully exclude publication bias. Supplementary Materials report selection-model and PET-PEESE sensitivity checks, which indicate some attenuation under bias-adjusted models but do not fully nullify the positive pooled signal.

### 3.5. Leave-One-Out Sensitivity Analysis

The overall conclusions were maintained with the removal of any single study. The pooled estimates ranged from *g* = 0.462 to *g* = 0.678 (original *g* = 0.542), with 95% Cis excluding 0 across all 24 permutations (minimum CI lower bound = 0.344; Table 5). Three studies showed high influence (|Δ*g*| ≥ 0.05): Study 7 (Park et al., 2019 [33]; *k* = 24, 18% of total *k*), in which removal increased g to 0.678 (Δ*g* = +0.137); Study 14 (Lee, Y.J., 2020 [40]; *k* = 11), in which removal yielded the most conservative estimate (g = 0.462, 95% CI: 0.344–0.580, Δ*g* = −0.079), and Study 16 (Jeong, S.R., 2019 [42]; *k* = 4), in which removal decreased g to 0.472 (Δ*g* = −0.069). The remaining 18 studies (75%) showed minimal individual influence (|Δ*g*| range: 0.001–0.020), suggesting that the pooled conclusion was not driven by a small number of individual studies.

### 3.6. Subgroup Analyses

For the 14 categorical moderator analyses, a standard random-effects model (*k* = 128) was pragmatically maintained for exploratory purposes to ensure model convergence and prevent critical degrees-of-freedom deficiencies. The independent-effects DerSimonian–Laird model was run (*k* = 128) for comparability with prior literature and as a sensitivity check.

Fourteen categorical moderators in Table 6 were examined in exploratory subgroup analyses without formal multiple-comparison correction; therefore these subgroup findings should be regarded as hypothesis-generating. Subgroups with very small *k* (e.g., meditation-based, *k* < 5) are particularly imprecise and vulnerable to performance/detection bias where participant blinding is infeasible. Large subgroup estimates (e.g., meditation *g* ≈ 2.490) require confirmatory randomized trials before any comparative claims are made.

NRCTs (g = 0.709) yielded larger pooled effect sizes than RCTs (g = 0.479), which may reflect confounding, selection effects, or other design-related differences rather than a true superiority of non-randomized studies.

By program location, forest bathing areas (*g* = 1.326) and healing forests (*g* = 1.092) had large and statistically significant effect sizes.

Regarding program participation format, residential (overnight) programs (*g* = 0.862) had markedly larger effect sizes than non-residential (no-lodging) programs (*g* = 0.448).

Meditation-centered interventions demonstrated the largest effects among all intervention types (*g* = 2.490), a magnitude that substantially exceeded exercise-based formats (*g* = 0.343) and suggests the theoretical possibility that integrating structured mindfulness practices within forest environments may activate ART–SRT pathways; however, this differential should not be interpreted as evidence of superiority over exercise-based formats, given the limited number of contributing studies and the structural impossibility of blinding.

Given the very small number of contributing studies, the precision of this estimate is inherently limited; this pattern is noted solely as a hypothesis-generating signal and does not support claims of superior efficacy.

Residential programs also showed larger pooled effect sizes than same-day session formats (*g* = 0.862 vs. *g* = 0.448), a pattern consistent with the ART ‘being away’ mechanism; however, potential confounding from differences in total forest exposure time, program intensity, and participant self-selection cannot be excluded.

### 3.7. Certainty of Evidence

The GRADE framework was used to assess the certainty of evidence for the primary outcome. The overall certainty for the effect of forest therapy on middle-aged Korean women’s health (*g* = 0.596/0.542) was rated as “Low” on the basis of two downgraded domains: (1) risk of bias (−1): lack of detailed reporting on allocation concealment and the structural impossibility of blinding in field-based interventions, and (2) inconsistency (−1): very high statistical heterogeneity (*I*^2^ = 75.1%) and a wide prediction interval.

Although Egger’s regression and Begg–Mazumdar rank correlation tests indicated statistically significant funnel plot asymmetry (*p* < 0.05), the publication bias domain was rated as “no serious concern” and was not downgraded, consistent with the GRADE guidelines that downgrading requires evidence that missing studies would substantively alter the pooled conclusion. This condition was not met considering the Rosenthal Fail-safe N of 11,511 (tolerance threshold: 650).

The indirectness domain was not applied as a downgrade factor. The systematic search identified 36 studies meeting the systematic review criteria, of which two international studies were excluded from the meta-analysis owing to the absence of a comparison group. Thus, all 24 meta-analyzed studies originated in Korea. Korean middle-aged women were defined as the target population of this meta-analysis before it was conducted (Section 3.1), and all 24 included studies were drawn from the same population. Accordingly, the findings possess high directness concerning the target population (Korean middle-aged women), precluding the need for a downgrade due to population-level indirectness.

Although Section 4.4 addresses international generalizability concerns as substantive limitations, they do not constitute a GRADE indirectness penalty when the research question and evidence base are co-aligned. However, the certainty for meditation-based interventions was rated as “Moderate,” given the exceptionally large and consistent effect size (*g* = 2.490), which partially offsets the inconsistency domain. This upgrading was applied on the basis of a very large effect size (*g* = 2.490) meeting the GRADE upgrading criterion for large magnitude, consistent with Guyatt et al. (2008) [51] guidelines; however, given the small number of contributing studies and the structural impossibility of blinding, this rating should be interpreted with commensurate caution.

The “Low” overall GRADE rating does not imply that forest therapy is ineffective for Korean middle-aged women. Instead, it indicates that further high-quality RCTs are required before confident recommendations can be made and that international evidence remains absent, representing a critical gap for future research.

## 4. Discussion

This meta-analysis demonstrated a primary pooled Hedges’ *g* = 0.596 (SE = 0.081, 95% CI: 0.432–0.760, three-level RVE) and a supplementary *g* = 0.542 (95% CI: 0.420–0.664, standard RE model), suggesting a positive effect of forest therapy on health-related outcomes among middle-aged Korean women. Although direct comparisons with pharmacotherapy are methodologically complex and beyond the scope of this review, this magnitude is consistent with the effect sizes reported in prior Korean forest therapy evidence [31,52].

These findings remained robust in analyses restricted to RCTs (*g* = 0.479) and across all 24 leave-one-out permutations (*g* range: 0.462–0.678, all CIs excluding 0). The 95% prediction interval (PI: −0.721 to 1.913) underscores substantial context-dependency. Outcomes in a new, unstudied setting may be negligible, depending on protocol fidelity, underscoring the importance of adhering to the high-efficacy formats identified in subgroup analyses of residential and meditation-based programs.

Program type largely accounted for the confirmed high heterogeneity (*I*^2^ = 75.1%). Forest bathing areas (*g* = 1.326) and healing forests (*g* = 1.092), meditation-based programs (*g* = 2.490), and residential programs led by certified instructors (*g* = 0.862) were associated with larger pooled effect sizes in exploratory subgroup analyses (uncorrected for multiple comparisons); these estimates should not be interpreted as confirmed superiority signals. The indoor environment subgroup (which includes exercise-based and indirect programs) showed a modest but significant effect (*g* = 0.377). These findings suggest that program type and environmental context may be important factors associated with variation in therapeutic outcomes.

Among the 14 moderator variables examined, two categorical variables produced the largest between-group Q-statistics, thereby accounting for the greatest portion of the observed heterogeneity: intervention type (*Q* = 132.9, *p* < 0.001) and forest environment category (*Q* = 50.777, *p* < 0.001). This pattern is consistent with the hypothesis that program type and environmental context may be important sources of variability in therapeutic outcomes—specifically, what participants do in the forest (meditation vs. exercise vs. indirect exposure) and where they do it (designated healing-forest infrastructure vs. general woodland); however, the exploratory nature of these moderator analyses precludes definitive conclusions about causal drivers of heterogeneity.

The remaining heterogeneity that is unaccounted for by these moderators likely reflects the true variability in participant characteristics, facilitator expertise, and site-specific microclimate factors that the available data cannot fully disentangle, further highlighting the need for standardized future trials.

### 4.1. Biopsychosocial Mechanism 1: Residential Immersion and Attentional Recovery Through the ART “Being Away” Mechanism

One theoretical interpretation, consistent with the ART ‘being away’ mechanism, is that physical separation from domestic environments may be an active ingredient not replicable by extended same-day sessions; however, this hypothesis requires confirmation in adequately powered RCTs that directly compare matched-duration residential and non-residential formats before any program substitution claims can be made. That said, this subgroup comparison is subject to uncontrolled confounds including differences in total forest exposure time, program intensity, and participant self-selection, which preclude causal attribution. Program planners and policymakers should treat this finding as preliminary justification for prioritizing residential format investigation in future RCTs rather than as definitive evidence for program expansion.

### 4.2. Biopsychosocial Mechanism 2: Meditation-Enhanced Attentional and Physiological Restoration (ART–SRT Synergy)

The large effect size of meditation-based interventions (*g* = 2.490 vs. exercise *g* = 0.343) suggests the theoretical possibility that integrating structured mindfulness practices within forest environments may activate ART-SRT pathways; however, this differential should not be interpreted as evidence of superiority over exercise-based formats, given the limited number of contributing studies and the structural impossibility of blinding.

The extremely large effect size (*g* = 2.490) may partly reflect performance or detection bias inherent to small-scale field trials in which participant blinding is structurally impossible, and the contributing study pool is insufficiently large to rule out chance findings or publication artifacts. This pattern is consistent with the synergistic activation of two established theoretical pathways.

Specifically, ART proposes that involuntary fascination stimuli provided by natural environments reduce cognitive load and liberate resources for higher-order reflective processing [12], while SRT posits that parasympathetic activation in restorative environments reduces the physiological arousal that ordinarily interferes with metacognitive processing [11]. Structured meditation in a forest setting may amplify both pathways simultaneously, thereby enabling a depth of psychological processing that exercise-based formats alone may not elicit to the same degree, though this differential remains an empirical hypothesis requiring confirmatory evidence.

If confirmed in future trials, the forest may function not only as “a place to walk” but also as a context for sustained reflective attention. The exceptional magnitude of this effect (*g* = 2.490) merits both recognition and contextually appropriate interpretation. The contributing studies were predominantly small-scale field-based trials in which participant blinding was structurally impossible—a limitation inherent to virtually all experiential forest therapy research. This constraint prevents causal inference, and the ART–SRT framework should be understood as providing a theoretical account consistent with the observed pattern rather than confirming it.

Accordingly, this finding should be treated as an exploratory signal of substantive magnitude warranting prioritized replication in adequately powered independent trials, rather than dismissed as a methodological artifact. Adequately powered, independently conducted randomized trials are warranted to establish a confirmatory evidence base, but the present pattern provides a theoretical rationale for prioritizing meditation-based formats in future confirmatory research designs, while implementation in actual programs should await RCT-level confirmation.

### 4.3. Biopsychosocial Mechanism 3: Limitations in Addressing Climacteric Physical Symptoms

The effect size for the climacteric symptoms group did not reach statistical significance (*g* = 0.196, *p* = 0.059) [53]. Therefore, forest therapy cannot be recommended as a standalone definitive intervention for this subgroup at present, necessitating further heavily powered trials.

### 4.4. Limitations and Future Directions

The following limitations should be considered when interpreting the current findings. Although this review provided quantitative evidence through systematic integration of controlled studies, several methodological constraints limit the scope of its conclusions. First, the methodological rigor of future research warrants improvement.

Considering that most prior studies were biased toward non-randomized designs with confirmed bias potential and high statistical heterogeneity, future research should prioritize randomized controlled designs (RCTs) and establish standardized research protocols. Second, long-term follow-up and expanded utilization of physiological indicators would be warranted to complement the current findings. Third, differentiated research that considers participant characteristics would be warranted to reflect diverse variables in middle-aged Korean women, including age, climacteric status, and socioeconomic background. Fourth, the higher pooled effect observed in NRCTs than in RCTs (*g* = 0.709 vs. *g* = 0.479) is consistent with confounding by indication, whereby participants in studies using non-randomized designs may be systematically more motivated or self-selected than those in randomized trials, rather than reflecting a genuine superiority of non-randomized designs. Therefore, NRCT results should be interpreted with additional caution. Fifth, the psychological and physical health outcomes synthesized in this review (e.g., depression, anxiety, stress, and insomnia) reflect self-reported symptom-scale scores rather than formal clinical diagnoses based on psychiatric criteria such as the DSM-5. Consequently, the baseline generalizability to clinical psychiatric populations remains limited, and future trials should explicitly incorporate validated diagnostic tools alongside objective physiological biomarkers. Sixth, although the 128 effect sizes nested within 24 studies present potential statistical dependency concerns, this review proactively addressed these through multiple complementary analytical approaches: leave-one-out analyses for the full *k* = 128 dataset and a primary three-level random-effects model with robust variance estimation (RVE, CR2 correction), with the DerSimonian–Laird model serving as a supplementary sensitivity check. Nevertheless, the subgroup confidence intervals in the primary analysis may still underestimate true inferential uncertainty, and future independent replications are encouraged to apply these methods as primary rather than supplementary analytical frameworks. Seventh, and finally, the external validity of these findings warrants careful consideration. All 24 controlled studies were conducted in the Republic of Korea, a country with a nationally standardized healing forest infrastructure. Whether the documented effects generalize to ecologically divergent settings—boreal coniferous forests, tropical rainforests, or semi-arid woodlands—remains an open question, as forest microclimate parameters differ substantially across biomes and may modulate both physiological stress recovery and the attentional-restoration pathways identified here. Moreover, the cultural practice of forest bathing formalized through national legislation, may constitute a therapeutic ingredient that is not readily transferable to contexts lacking equivalent institutional recognition. Future international research employing cross-national designs, standardized environmental characterization protocols, and culturally adapted outcome measures is needed to determine which components of the therapeutic benefits of forest therapy are universal and which are context-specific.

### 4.5. Preliminary, Hypothesis-Generating Considerations for Forest Therapy Program Development in Middle-Aged Korean Women’s Health Promotion (GRADE: Low)

Given that the overall certainty of evidence is rated ‘Low’ by GRADE, the actionable guidelines originally presented in Table 7 have been strictly reframed as preliminary, hypothesis-generating considerations for future research and pilot program design, rather than as definitive clinical or policy recommendations.

From a public health perspective, these exploratory findings are most directly applicable to three service contexts: community mental health programs targeting the menopausal transition, worksite health promotion for middle-aged Korean women managing multiple caregiving roles, and health authority-supported wellness initiatives in nations with an established healing-forest infrastructure. Integrating these findings into healthcare systems does not necessitate a wholesale adoption of the South Korean national model; instead, stakeholders should focus on modifiable core elements associated with larger observed effect sizes in this synthesis, such as meditation-based content, qualified facilitation, and immersive natural environments.

To elevate the certainty of this evidence base, future research may productively focus on conducting high-quality, adequately powered randomized controlled trials (RCTs) of meditation-centered formats, piloting standardized short-intensive residential protocols under formal evaluation, and systematically testing climacteric-specific forest therapy protocols.

## 5. Conclusions

This systematic review and meta-analysis suggests that forest therapy may be associated with improvements in health-related outcomes among middle-aged Korean women (Hedges’ *g* = 0.596 (SE = 0.081, 95% CI: 0.432–0.760) [three-level RVE model]; supplementary DL model: *g* = 0.542, 95% CI: 0.420–0.664).

The overall GRADE certainty is Low, indicating that confidence in the effect estimate remains limited (risk of bias and inconsistency). Meditation-based interventions were rated Moderate; however, this subgroup finding should be interpreted cautiously because it was based on a limited number of studies and remains exploratory.

Although the number of contributing studies was limited and participant blinding was structurally impossible, the ART–SRT theoretical framework provides a plausible conceptual account for the observed pattern; however, this theoretical coherence cannot substitute for internal validity, and the finding must be treated strictly as an exploratory signal requiring confirmation in adequately powered independent trials.

Residential programs were similarly associated with larger exploratory effect sizes than same-day formats (*g* = 0.862 vs. 0.448), a finding consistent with the ART ‘being away’ mechanism; the practical magnitude of this differential (nearly half a standard deviation) and the theoretical grounding together provide preliminary justification for prioritizing residential format investigation in future RCTs, notwithstanding the exploratory nature of the subgroup and the uncontrolled confounds of total exposure time and participant self-selection.

The “Moderate” GRADE rating reflects the consistency and magnitude of the within-subgroup signal, not a determination that meditation-based forest therapy is definitively superior to other intervention formats; adequately powered, independently conducted randomized trials are required before any comparative claims can be made with confidence.

Accordingly, these findings should be treated as preliminary, hypothesis-generating evidence rather than as definitive confirmation of forest therapy as a complementary community health promotion strategy. The largest exploratory effect sizes were observed for meditation-based content, residential format, and purpose-designed healing forest settings; independent replication is required before these patterns can be described as reliably consistent.

Given the ‘Low’ overall GRADE certainty rating, the implementation-oriented language in Section 4.1, Section 4.2 and Section 4.5 and Table 7 should be read strictly as hypothesis-generating and preliminary evidence-informed considerations—not as actionable clinical recommendations or policy directives.

In sum, despite methodological and design limitations in the primary studies, the three-level meta-analysis provides a coherent synthesis identifying promising program formats (e.g., meditation-enhanced and residential designs) warranting prioritized confirmatory trials. However, the evidence base is confined to controlled studies conducted in Korea, and the overall certainty is Low (GRADE) due to risk of bias and high heterogeneity. Therefore, these results should be interpreted as preliminary, hypothesis-generating evidence specific to middle-aged Korean women rather than as support for broad clinical or policy adoption. High-quality, preregistered, adequately powered RCTs—particularly independent international trials using standardized protocols—are required to confirm efficacy and support generalization beyond the Korean context.

## Figures and Tables

**Table 1:** PICOTS-SD Framework Definition.

Element	Category	Inclusion Criteria	Exclusion Criteria
P (Population)	Middle-aged women	Women aged 40–65 years; limited to Korean women following systematic search (see Section 3.1)	Men; mixed-gender without sex-stratified data; age outside 40–65
I (Intervention)	Forest therapy Programs	Instructor-led, researcher-designed, or indoor-based forest therapy programs	Urban parks only; non-forest NBI; pharmacological interventions
C (Comparator)	Comparison group	Waitlist control, usual care, or active control (meta-analysis only)	No comparator (systematic review only)
O (Outcomes)	Health effects	Mental (stress, depression, anxiety, quality of life (QoL)), physiological (cortisol, BP, NK cells), physical (fitness, BMI)	Non-health outcomes
T (Timeframe)	No restriction	Any duration; classified by session count and program type	—
S (Setting)	Population type	General population or symptomatic/disease groups	
SD (Study Design)	Study design	RCTs, NRCTs, single-group pre–post designs (latter excluded from meta-analysis)	Case reports, qualitative studies, reviews

**Table 2:** Overall Effect Size (Primary Three-Level RVE Model and Supplementary DL Model, *k* = 128 Effect Sizes, 24 Studies).

Model	Hedges’ *g*	SE	95% CI Lower	95% CI Upper	*z*-Value	*p*-Value
Primary three-level RVE model	0.596	0.081	0.432	0.760	7.36	<0.001
Random Effects Model (*k* = 128)	0.542	0.062	0.420	0.664	8.70	<0.001

Note. The statistical parameters of the primary three-level RVE model can be verified in the Supplementary Materials in Document S4 R software supplementary materials.

**Table 3:** Heterogeneity Statistics for Random-Effects Model (*k* = 128 Effect Sizes, 24 Studies).

*Tau*	*Tau* ^2^	*Q*-Value	*df*[Q]	*I* ^2^
0.575	0.332	509.71	127	75.1%

Note. *Tau* = between-study standard deviation; *Tau*^2^ = variance; *Q*-value = Cochran’s *Q*; *df*(*Q*) = 127; *I*^2^ = proportion of variance due to heterogeneity. Heterogeneity and publication bias diagnostics were performed based on the standard random-effects framework (*k* = 128) to strictly evaluate the distribution and asymmetry across all extracted data points, which informed the exploratory moderator analyses.

**Table 4:** Publication Bias Assessment for *k* = 128.

Test	Statistic	*p*-Value	Missing Studies (Trim-Fill)	Fail-Safe N
Egger’s Regression Test	*t* = 3.42	<0.05	—	—
Begg–Mazumdar Rank Correlation	*τ* = 0.18	<0.05	—	—
Rosenthal Fail-Safe N	N = 11,511	Not Applicable	0	11,511

**Table 5:** Leave-One-Out Sensitivity Analysis: Pooled Hedges’ *g* After Systematically Removing Each Study (Original *g* = 0.542; 95% CI: 0.420–0.664). Note: Leave-one-out sensitivity analysis was conducted based on the supplementary standard random-effects model (*k* = 128) for computational compatibility.

Study #	*k*	Study Description	Hedges’ *g* (Removed)	SE	95% CI Lower	95% CI Upper	Δ*g*	Influence
High Influence |Δ*g*| ≥ 0.05 (*n* = 3)								
#7	24	Forest therapy stress management program	0.678	0.0831	0.5150	0.8408	+0.1370	High
#14	11	Phytoncide inhalation (mood, HRQOL; climacteric)	0.462	0.0601	0.3443	0.5799	−0.0788	High
#16	4	Forest experience program (climacteric psychological symptoms)	0.472	0.0572	0.3603	0.5846	−0.0685	High
Moderate Influence 0.02 ≤ |Δ*g*| < 0.05 (*n* = 3)								
#5	6	Forest therapy for psychological improvement in Korean middle-aged women	0.571	0.0670	0.4393	0.7020	+0.0297	Moderate
#11	8	Forest walking program (HRQOL, functional movement)	0.567	0.0636	0.4427	0.6920	+0.0264	Moderate
#17	3	Forest therapy program (stress reduction in middle-aged women)	0.499	0.0589	0.3836	0.6144	−0.0419	Moderate
Minimal Influence |Δ*g*| < 0.02 (*n* = 18)—Representative Examples								
#1	4	Expressive writing-based forest therapy (stress, depression)	0.548	0.0633	0.4237	0.6717	+0.0068	Minimal
#2	2	EFT program (climacteric symptoms, QoL)	0.538	0.0623	0.4160	0.6603	−0.0028	Minimal
#3	3	Forest therapy program (depression, stress, QoL)	0.522	0.0617	0.4009	0.6428	−0.0191	Minimal
#4	5	Indoor exercise + phytoncide diffusion	0.559	0.0632	0.4347	0.6826	+0.0177	Minimal
#6	5	8-week combined forest resistance exercise (breast cancer)	0.548	0.0627	0.4250	0.6708	+0.0070	Minimal
#8	2	Essential oil inhalation necklace (anxiety, depression)	0.544	0.0632	0.4201	0.6678	+0.0030	Minimal
#9	3	Forest terrain exercise + phytoncide (BDNF, IGF-1, VEGF)	0.536	0.0623	0.4141	0.6582	−0.0048	Minimal
#10	3	Forest therapy (stress, fatigue, depression)	0.521	0.0620	0.3998	0.6427	−0.0196	Minimal
#12	1	Forest physical activity (stress, vascular health)	0.524	0.0607	0.4045	0.6425	−0.0174	Minimal
#13	8	Forest walking exercise (antioxidant hormones, immune function)	0.554	0.0649	0.4271	0.6814	+0.0133	Minimal
#15	9	12-week forest walking (fitness, self-efficacy, stress)	0.559	0.0645	0.4328	0.6855	+0.0182	Minimal
#18	3	Barefoot forest trail walking effect	0.551	0.0628	0.4277	0.6741	+0.0100	Minimal
#19	3	REBT group counseling-based forest therapy (depression, self-esteem)	0.540	0.0629	0.4168	0.6635	−0.0008	Minimal
#20	3	Forest exercise (HDL-C, LDL-C, triglyceride changes)	0.553	0.0629	0.4296	0.6760	+0.0119	Minimal
#21	7	Forest exercise program (HRQOL, functional movement)	0.561	0.0637	0.4357	0.6855	+0.0197	Minimal
#22	5	12-week forest exercise (blood lipids, SOD, melatonin)	0.536	0.0625	0.4137	0.6586	−0.0047	Minimal
#23	2	12-week forest walking (fitness, self-efficacy)	0.539	0.0625	0.4167	0.6616	−0.0017	Minimal
#24	4	Fir essential oil aroma massage (stress, sleep disorders, fatigue)	0.527	0.0622	0.4050	0.6489	−0.0139	Minimal
Original Overall Estimate (all 24 studies)			0.542	0.0623	0.4200	0.6640		

Note. Abbreviations used in Table 5—HRQOL: Health-Related Quality of Life; EFT: Emotional Freedom Technique; REBT: Rational Emotive Behavior Therapy; BDNF: Brain-Derived Neurotrophic Factor; IGF-1: Insulin-like Growth Factor 1; VEGF: Vascular Endothelial Growth Factor; HDL-C: High-Density Lipoprotein Cholesterol; LDL-C: Low-Density Lipoprotein Cholesterol; SOD: Superoxide Dismutase; QoL: Quality of Life.

**Table 6:** Subgroup Random Effect Size Analysis.

Category	Sub-Group	*k*	Hedges’ *g*	Standard Error	95% CI Lower Limit	95% CI Upper Limit	*Q*-Value	*df*	*p*-Value
Study Type	Doctoral Dissertation	44	0.701	0.105	0.495	0.907	3.516	2	0.172
	Master’s	37	0.42	0.115	0.195	0.647			
	Journal	47	0.504	0.103	0.302	0.707			
Study Design	RCTs	88	0.479	0.075	0.332	0.627	2.858	1	0.091
	NRCTs	40	0.709	0.113	0.487	0.930			
Group Size	1~5	5	1.102	0.33	0.456	1.749	57.656 **	4	< 0.001
	6~10	45	0.070	0.096	−0.118	0.259			
	11~15	26	1.290	0.138	1.009	1.550			
	16~20	6	0.263	0.275	−0.276	0.803			
	21~25	46	0.647	0.099	0.453	0.840			
Health Condition	General	112	0.564	0.066	0.435	0.694	5.654	2	0.059
	Insomnia	5	1.102	0.342	0.432	1.772			
	Menopausal	11	0.196	0.204	−0.204	0.596			
Environment	General forest	54	0.205	0.088	0.031	0.378	50.777 **	3	< 0.001
	Forest bathing site	9	1.326	0.239	0.858	1.794			
	Indoor	30	0.377	0.114	0.153	0.602			
	Healing forest	35	1.092	0.115	0.867	1.317			
Program type	Indirect	15	0.080	0.177	−0.427	0.268	16.684 **	2	< 0.001
	Structured	39	0.776	0.112	0.557	0.995			
	Self-leading	74	0.557	0.080	0.399	0.714			
Intervention	Combined	13	0.159	0.144	−0.124	0.442	132.9 **	4	< 0.001
	Exercise-based	59	0.343	0.070	0.202	0.481			
	Exercise + Mental	27	0.871	0.110	0.655	1.087			
	Mental	12	2.490	0.201	2.095	2.884			
	Aroma (indirect indoor)	17	0.016	0.132	−0.244	0.276			
Measure Index	Physiological	24	0.542	0.138	0.271	0.813	15.464 **	5	0.009
	Physiological + Physical	5	1.102	0.337	0.441	1.763			
	Physical	15	0.166	0.175	−0.177	0.509			
	Physical + Psychological	9	0.145	0.204	−0.254	0.544			
	Psychological	45	0.559	0.105	0.353	0.765			
	Psycho+Physio	30	0.805	0.127	0.556	1.054			
Accommodation Format	Lodging	30	0.862	0.125	0.617	1.107	8.485 **	1	0.004
	No Lodging	98	0.448	0.068	0.315	0.581			
Duration	2 Weeks	44	0.654	0.106	0.448	0.861	45.88 **	5	< 0.001
	4 Weeks	9	0.937	0.239	0.468	1.405			
	6 Weeks	10	1.707	0.233	1.250	2.164			
	8 Weeks	11	0.196	0.199	−0.194	0.587			
	10 Weeks	26	0.046	0.133	−0.214	0.306			
	12 Weeks	28	0.530	0.124	0.287	0.773			
Times/Week	1 Time/Week	50	0.659	0.101	0.461	0.857	3.266	3	0.352
	2 Times/Week	20	0.651	0.165	0.326	0.975			
	3 Times/Week	56	0.429	0.093	0.247	0.611			
	Daily	2	0.471	0.480	−0.469	1.412			
XSessions	1~10	66	0.745	0.087	0.574	0.916	18.673 **	3	< 0.001
	11~20	20	0.431	0.149	0.140	0.722			
	21~30	23	0.043	0.142	−0.236	0.322			
	31~40	19	0.642	0.153	0.342	0.941			
Hours/Sessions	< 2 h	85	0.573	0.077	0.423	0.723	5.416	4	0.247
	2–4 h	2	0.471	0.475	−0.460	1.402			
	4–6 h	6	0.261	0.287	−0.302	0.825			
	6–8 h	24	0.713	0.143	0.433	0.994			
	No mention	11	0.196	0.205	−0.205	0.597			
Effect point	Physiological	30	0.593	0.128	0.342	0.845	5.446	3	0.142
	Physical	26	0.273	0.135	0.008	0.538			
	Psychological	71	0.640	0.085	0.473	0.807			
	Cognitive	1	0.593	0.666	−0.713	1.899			

Note. ** *p* < 0.001; *k* = number of effect sizes; *Q*-value = *Q* statistic; *df* = degree of freedom. All subgroup analyses are exploratory; no multiple-comparison correction was applied (Bonferroni-adjusted threshold: *p* < 0.004). The meditation-based subgroup (*g* = 2.490) involves a small number of contributing studies and structurally impossible blinding; interpret with particular caution.

**Table 7:** Preliminary Evidence-Informed Considerations for Forest Therapy Program Development in Middle-Aged Women’s Health Promotion (Overall GRADE Certainty: Low; for research planning and hypothesis generation only).

Program Element	Recommendation	Evidence	Theoretical Mechanism	Implementation Notes
Intervention Type	Meditation-centered content	*g* = 2.490 vs. exercise *g* = 0.343	ART + SRT synergy: involuntary fascination reduces cognitive load; parasympathetic activation reduces arousal	Mindfulness-based forest activities; sensory awareness practice
Setting	Designated healing forest or forest bathing area	*g* = 1.326/1.092 vs. general trails *g* = 0.205	Biophilia + 4 qualities of restorative environments	Purpose-designed infrastructure essential; urban parks inappropriate
Program Format	Residential (≥1 night)	*g* = 0.862 vs. same-day *g* = 0.448	ART ‘being away’ mechanism (residential format enables full attentional recovery)	Physical separation from home environment essential
Duration	≤10 sessions (intensive format)	Short intensive programs superior to long low-intensity	Concentrated recovery exposure principle	High adherence in short intensive format
Group Size	11–15 participants	*g* = 1.290 (highest in group size category)	Balance of social support and personal space	Avoid both isolation (<5) and overcrowding (>40)
Facilitator	Certified forest therapy instructor	Certification associated with program fidelity	Expert facilitation of structured mindfulness and reflective attention practices	Certified expertise in both forest ecology and mindfulness-based psychological facilitation is highly recommended; where national certification systems do not yet exist, structured training programs covering both domains are strongly advised; urban park facilitators without specialist training are unlikely to achieve equivalent outcomes
Special Population Notes	Climacteric subgroup: integrate SSFT components	Standard FT *g* = 0.196 in climacteric group	Symptom-specific protocols needed for physical symptoms	Standard forest therapy protocols produced non-significant effects in climacteric-specific subgroups (*g* = 0.196, *p* = 0.059), suggesting the need for symptom-targeted program components. Future protocol development should be informed by future RCTs.

Note. SSFT: Symptom-Specific Forest Therapy, Standard FT: Standard Forest Therapy, (*ns*): not significant. All items in this table represent exploratory, hypothesis-generating considerations derived from ‘Low’ overall GRADE certainty evidence.

## Data Availability

The coding sheet (Excel) and full analysis dataset are available as Supplementary Materials and upon reasonable request to the corresponding author, in accordance with MDPI data transparency policy. The data presented in this study are available on request from the corresponding author.

## Supplementary Materials

The following supporting information can be downloaded at: https://www.mdpi.com/article/10.3390/healthcare14111569/s1. Table S1: Effect Level Data, Document S1: Retrospective_Protocol Summary INPLASY final, Document S2: analysis 3level, Document S3: compute effects, Document S4: analysis RVE sensitivity, Document S5: R SessionInfo.

## Appendix A

**Table A1:** Included studies for meta-analysis.

#	Author, Year	Design	n (Exp)	n (Con)	Age (years)	Setting	Format	Duration	Freq/Wk	Sessions	Outcome	Hedges’ *g*
1	Kim, A.R., 2024 [27]	NRCT	12	12	40–59	Forest bathing site	Non-residential	6 wks	2/wk	12 sess.	Psychological	−0.146
2	Kim, H.G., 2016 [28]	RCT	8	6	55 ± 2.01	Healing forest	Non-residential	6 wks	1/wk	6 sess.	Psychological	1.166
3	Kim, H.S., 2018 [29]	NRCT	7	7	55.4 (±2.0)	Healing forest	Non-residential	4 wks	1/wk	4 sess.	Psychological	3.157
4	Min, J.W., 2023 [30]	RCT	12	12	54.25 ± 3.11	Indoor	Non-residential	12 wks	3/wk	36 sess.	Physiological	0.595
5	Park, B.J., 2022 [31]	RCT	53	53	mean 53	Healing forest	Residential	2 wks	3/wk	6 sess.	Psychological	0.163
6	Park, J.S., 2023 [32]	NRCT	6	6	50.33 ± 7.53	General forest	Non-residential	2 wks	2/wk	4 sess.	Physiological	−0.485
7	Park, C.E., 2019 [33]	RCT	53	53	mean 53	Healing forest	Residential	2 wks	1/wk	2 sess.	Physiological	0.392
8	Shin, D.J., 2023 [34]	NRCT	37	34	40–65	Indoor	Non-residential	2 wks	7/wk	14 sess.	Psychological	0.479
9	Shin, M.J., 2016 [35]	RCT	11	10	50–59	General forest	Non-residential	12 wks	3/wk	36 sess.	Physiological	1.654
10	Oh, J.Y., 2015 [36]	NRCT	20	20	48.40 ± 8.34	General forest	Non-residential	4 wks	1/wk	4 sess.	Psychological	1.289
11	Yoo, S.R., 2015 [37]	RCT	7	7	54–64	General forest	Non-residential	10 wks	3/wk	30 sess.	Physical	0.074
12	Yoon, M.A., 2020 [38]	NRCT	10	10	51–58	General forest	Non-residential	12 wks	1/wk	12 sess.	Psychological	4.241
13	Lee, M.O., 2018 [39]	NRCT	10	10	51–57	General forest	Non-residential	12 wks	3/wk	36 sess.	Physiological	0.181
14	Lee, Y.J., 2020 [40]	RCT	20	21	53.4 ± 2.68	Indoor	Non-residential	8 wks	1/wk	8 sess.	Psychological	0.593
15	Lee, J.S., 2016 [41]	RCT	11	9	59.32 ± 7.11	General forest	Non-residential	12 wks	3/wk	36 sess.	Physical	−0.275
16	Jeong, S.R., 2019 [42]	NRCT	11	11	50–60	Forest bathing site	Non-residential	6 wks	2/wk	12 sess.	Psychological	4.400
17	Jeong, Y.G., 2023 [43]	RCT	20	20	40s–50s	General forest	Non-residential	2 wks	1/wk	2 sess.	Psychological	4.368
18	Jeong, H.J., 2023 [44]	NRCT	9	9	40–64	General forest	Non-residential	4 wks	2/wk	8 sess.	Psychological	0.363
19	Choi, G., 2023 [45]	NRCT	11	11	40–65	General forest	Non-residential	10 wks	1/wk	10 sess.	Psychological	0.449
20	Choi, J.H., 2010 [46]	NRCT	10	10	45–54	General forest	Non-residential	10 wks	3/wk	30 sess.	Physiological	−0.186
21	Choi, J.H., 2018 [47]	RCT	11	9	56.57 ± 4.0	General forest	Non-residential	10 wks	3/wk	30 sess.	Physical	−0.275
22	Choi, J.H., 2017 [48]	RCT	12	12	52.37 ± 2.43	General forest	Non-residential	10 wks	3/wk	30 sess.	Physiological	0.175
23	Choi, J.H., 2016 [49]	RCT	11	9	53.9 ± 2.69	General forest	Non-residential	12 wks	3/wk	36 sess.	Psychological	0.462
24	Hong, J.S., 2019 [50]	RCT	10	10	40s–50s	Indoor	Non-residential	2 wks	2/wk	4 sess.	Psychological	0.821

Notes: RCT, Randomized controlled trial; NRCT, Non-randomized controlled trial; Exp, Experimental group; Con, Control group. n = number of completed participants. Hedges’ *g* reflects the pooled representative effect size per study (total *k* = 128 independent effect sizes used in the meta-analysis). All studies: Korean domestic publications. Full bibliographic citations for all 24 included studies are provided in the Supplementary coding sheet (Table S1) in accordance with PRISMA 2020 Item 17 requirements. Each study is uniquely identified by Author and Year in this table.

**Table A2:** Search Engine and Results.

Search Engine	Key Word and Search Query	Result
Scopus	(“forest” OR “forest healing” OR “forest therap*” OR “forest bathing” OR “forest activity” OR “forest experience” OR “forest adventure” OR “nature” OR “natural activity” OR “shinrin-yoku”) AND (“middle-aged wom*n” OR “menopausal”)	694
PubMED	(“forest”[Title/Abstract] OR “forest healing”[Title / abstract] OR “forest therap*”[Title/abstract] OR “forest bathing”[Title/abstract] OR “forest activity”[Title/Abstract] OR “forest experience”[Title/Abstract] OR “forest adventure”[Title/abstract] OR “nature”[Title/Abstract] OR “natural activity”[Title/abstract] OR “shinrin-yoku” [Title/Abstract]) AND (“middle-aged wom*n”[Title/ Abstract] OR “menopausal”[Title/Abstract])	492
MEDLINE	TI(“forest” OR “forest healing” OR “forest therap*” OR “forest bathing” OR “forest activity” OR “forest experience” OR “forest adventure” OR “nature” OR “natural activity” OR “shinrin-yoku”) AND TI (“middle-aged wom*n” OR “menopausal”)	1794
Web of Science	(forest* OR “forest healing” OR “forest therap*” OR “forest bathing” OR “shinrin-yoku” OR “nature-based”) AND (“middle-aged women” OR menopaus* OR climacteric)	602
EMBASE	(‘forest’ OR ‘forest healing’ OR ‘forest therap*’ OR ‘forest bathing’ OR ‘forest activity’ OR ‘forest experience’ OR ‘forest adventure’ OR ‘nature’ OR ‘natural activity’ OR ‘shinrin-yoku’):ti,ab AND (‘middle-aged wom*n’ OR ‘menopausal’):ti,ab	1924
RISS	초록(Abstract):숲(forest)│숲 체험(forest experience)│숲 활동(forest activity)│숲놀이(forest play)│자연(nature)│산림(mountain forest)│도시숲(urban forest)│삼림욕(forest bathing)│산림테라피(forest therapy) <AND> 초록(Abstract):중년여성(middle-aged women)│갱년기(climacteric)│폐경기(menopause)	1965
DBpia	전체(All fields) = 숲(forest)│숲 체험(forest experience)│숲 활동(forest activity)│숲놀이(forest play)│자연(nature)│산림(mountain forest)│도시숲(urban forest)│삼림욕(forest bathing)│산림테라피(forest therapy) AND 전체(All fields)=중년여성(middle-aged women)│갱년기(climacteric)│폐경기(menopause)	545
KISS	전체(All fields) = “숲(forest)│숲 체험(forest experience)│숲 활동(forest activity)│숲놀이(forest play)│자연(nature)│산림(mountain forest)│도시숲(urban forest)│삼림욕(forest bathing)│산림테라피(forest therapy)” and 전체(All fields)=“중년여성(middle-aged women)│갱년기(climacteric)│폐경기(menopause)”	514
ScienceOn	초록(Abstract) = 숲(forest)│숲 체험(forest experience)│숲 활동(forest activity)│숲놀이(forest play)│자연(nature)│산림(mountain forest)│도시숲(urban forest)│삼림욕(forest bathing)│산림테라피(forest therapy) AND 초록(Abstract)=중년여성(middle-aged women)│갱년기(climacteric)│폐경기(menopause)	404
National Library	숲(forest)│숲 체험(forest experience)│숲 활동(forest activity)│숲 놀이(forest play)│자연(nature)│산림(mountain forest)│도시숲(urban forest)│삼림욕(forest bathing)│산림테라피(forest therapy) AND 중년여성(middle-aged women)│갱년기(climacteric)│폐경기(menopause)	610
Hand-searching		19
Total		9563

Note. The asterisk (*) acts as a truncation tool (or wildcard) that searches for the root word alongside all of its possible endings or variations.

**Table A3:** PRISMA (Preferred Reporting Items for Systematic review and Meta-Analysis Protocols) 2020 checklist.

No.	Section	Topic	Checklist Item (PRISMA 2020)	Reported	Location
**TITLE**					
1	Title	**Title**	Identify the report as a systematic review.	Yes	Title page
**ABSTRACT**					
2	Abstract	**Abstract**	See the PRISMA 2020 for Abstracts checklist.	Yes	Abstract
**INTRODUCTION**					
3	Introduction	**Rationale**	Describe the rationale for the review in the context of existing knowledge.	Yes	Section 1
4		**Objectives**	Provide an explicit statement of the objective(s) or question(s) the review addresses.	Yes	Section 1
**METHODS**					
5	Methods	**Eligibility criteria**	Specify the inclusion and exclusion criteria for the review and how studies were grouped for the syntheses.	Yes	Section 2.1; Table 1
6		**Information sources**	Specify all databases, registers, websites, organisations, reference lists and other sources searched or consulted to identify studies. Specify the date when each source was last searched or consulted.	Yes	Section 2.2; Table A2
7		**Search strategy**	Present the full search strategies for all databases, registers and websites, including any filters and limits used.	Yes	Section 2.2; Table A2
8		**Selection process**	Specify the methods used to decide whether a study met the inclusion criteria of the review, including how many reviewers screened each record and each report retrieved, whether they worked independently, and if applicable, details of automation tools used in the process.	Yes	Section 2.3
9		**Data collection process**	Specify the methods used to collect data from reports, including how many reviewers collected data from each report, whether they worked independently, any processes for obtaining or confirming data from study investigators, and if applicable, details of automation tools used in the process.	Yes	Section 2.3
10a		**Data items**	List and define all outcomes for which data were sought. Specify whether all results that were compatible with each outcome domain in each study were sought (e.g., for all measures, time points, analyses), and if not, the methods used to decide which results to collect.	Yes	Section 2.1; Table 1
10b		**Data items**	List and define all other variables for which data were sought (e.g., participant and intervention characteristics, funding sources). Describe any assumptions made about any missing or unclear information.	Yes	Section 2.1; Table 1
11		**Study risk of bias assessment**	Specify the methods used to assess risk of bias in the included studies, including details of the tool(s) used, how many reviewers assessed each study and whether they worked independently, and if applicable, details of automation tools used in the process.	Yes	Section 2.4
12		**Effect measures**	Specify for each outcome the effect measure(s) (e.g., risk ratio, mean difference) used in the synthesis or presentation of results.	Yes	Section 2.5
13a		**Synthesis methods**	Describe the processes used to decide which studies were eligible for each synthesis (e.g., tabulating the study intervention characteristics and comparing against the planned groups for each synthesis).	Yes	Section 2.5
13b		**Synthesis methods**	Describe any methods required to prepare the data for presentation or synthesis, such as handling of missing summary statistics, or data conversions.	Yes	Section 2.5
13c		**Synthesis methods**	Describe any methods used to tabulate or visually display results of individual studies and syntheses.	Yes	Section 2.5; Figure 3
13d		**Synthesis methods**	Describe any methods used to synthesize results and provide a rationale for the choice(s). If meta-analysis was performed, describe the model(s), method(s) to identify the presence and extent of statistical heterogeneity, and software package(s) used.	Yes	Section 2.5; Supplementary Materials
13e		**Synthesis methods**	Describe any methods used to explore possible causes of heterogeneity among study results (e.g., subgroup analysis, meta-regression).	Yes	Section 2.5; Section 3.6
13f		**Synthesis methods**	Describe any sensitivity analyses conducted to assess robustness of the synthesized results.	Yes	Section 2.5; Section 3.5
14		**Reporting bias assessment**	Describe any methods used to assess risk of bias due to missing results in a synthesis (arising from reporting biases).	Yes	Section 2.5; Section 3.4
15		**Certainty assessment**	Describe any methods used to assess certainty (or confidence) in the body of evidence for an outcome.	Yes	Section 2.5; Section 3.7
**RESULTS**					
16a	Results	**Study selection**	Describe the results of the search and selection process, from the number of records identified in the search to the number of studies included in the review, ideally using a flow diagram.	Yes	Section 3.1; Figure 1
16b		**Study selection**	Cite studies that might appear to meet the inclusion criteria, but which were excluded, and explain why they were excluded.	Yes	Section 3.1
17		**Study characteristics**	Cite each included study and present its characteristics.	Yes	Appendix A; Table A1
18		**Risk of bias in studies**	Present assessments of risk of bias for each included study.	Yes	Section 3.2; Figure 2
19		**Results of individual studies**	For all outcomes, present, for each study: (a) summary statistics for each group (where appropriate) and (b) an effect estimate and its precision (e.g., confidence/credible interval), ideally using structured tables or plots.	Yes	Figure 3 (Forest Plot); Supplementary Table S1 (n, Mean, SD per group for all 24 studies)
20a		**Results of syntheses**	For each synthesis, briefly summarise the characteristics and risk of bias among contributing studies.	Yes	Section 3.3, Section 3.4, Section 3.5 and Section 3.6
20b		**Results of syntheses**	Present results of all statistical syntheses conducted. If meta-analysis was done, present for each the summary estimate and its precision and measures of statistical heterogeneity. If comparing groups, describe the direction of the effect.	Yes	Table 2 and Table 3; Figure 3
20c		**Results of syntheses**	Present results of all investigations of possible causes of heterogeneity among study results.	Yes	Section 3.6; Table 6
20d		**Results of syntheses**	Present results of all sensitivity analyses conducted to assess the robustness of the synthesized results.	Yes	Section 3.5; Table 5
21		**Reporting biases**	Present assessments of risk of bias due to missing results (arising from reporting biases) for each synthesis assessed.	Yes	Section 3.4; Table 4; Figure 4
22		**Certainty of evidence**	Present assessments of certainty (or confidence) in the body of evidence for each outcome assessed.	Yes	Section 3.7
**DISCUSSION**					
23a	Discussion	**Discussion**	Provide a general interpretation of the results in the context of other evidence.	Yes	Section 4
23b		**Discussion**	Discuss any limitations of the evidence included in the review.	Yes	Section 4.4
23c		**Discussion**	Discuss any limitations of the review processes used.	Yes	Section 4.4
23d		**Discussion**	Discuss implications of the results for practice, policy, and future research.	Yes	Section 4.5; Table 7
**OTHER INFORMATION**					
24a	Other Information	**Registration and protocol**	Provide registration information for the review, including register name and registration number, or state that the review was not registered.	Yes	INPLASY (INPLASY202650156; registered 28 May 2026)
24b		**Registration and protocol**	Indicate where the review protocol can be accessed, or state that a protocol was not prepared.	Yes	Supplementary Document S1
24c		**Registration and protocol**	Describe and explain any amendments to information provided at registration or in the protocol.	N/A	Minor deviations from the registered protocol (INPLASY202650156): (1) actual search extended to February 2025 (registered: December 2024); (2) age range used 40–65 years (registered: 40–64); (3) CMA v4.0 used (registered: v3.0). These deviations did not affect primary outcomes.
25		**Support**	Describe sources of financial or non-financial support for the review, and the role of the funders or sponsors in the review.	Yes	Funding Statement
26		**Competing interests**	Declare any competing interests of review authors.	Yes	Conflicts of Interest
27		**Availability of data, code and other materials**	Report which of the following are publicly available and where they can be found: template data collection forms; data extracted from included studies; data used for all analyses; analytic code; any other materials used in the review.	Yes	Supplementary Materials; Data Availability

Yes = fully reported; N/A = not applicable. Items 24a–24c and Item 19 updated: INPLASY registration completed 28 May 2026 (INPLASY202650156; Item 19 Location updated to reference Supplementary Table S1 (Excel coding sheet, contains n, Mean, SD per group). PRISMA 2020 [54].
