# Supplementary material for: Validating the Effectiveness of Forest Therapy Programs for Middle-Aged Korean Women: A Systematic Review and Meta-Analytic Approach
Source: Healthcare (Basel). 2026 Jun 3;14(11):1569. doi: 10.3390/healthcare14111569 (PMC13257257; doi:10.3390/healthcare14111569)
Supplement: Supplementary file 1 [file healthcare-14-01569-s001.zip › Document_S1_ Retrospective_Protocol_Summary_INPLASY_final.pdf]

Protocol Summary (INPLASY Registration Completed: INPLASY202650156)

Validating the Effectiveness of Forest Therapy Programs for Middle-Aged Women: A Systematic Review and Meta-Analytic Approach

Healthcare (MDPI) | Young-Ho Lee, Gyeong-Min Min, Pyeong-Sik Yeon

This document provides a protocol summary documenting the key methodological decisions that were finalized prior to data extraction, consistent with PRISMA 2020 transparency standards. This review has been registered in INPLASY (International Platform of Registered Systematic Review and Meta-analysis Protocols; registration number: INPLASY202650156; doi: 10.37766/inplasy2026.5.0156; registration date: May 28, 2026).

1. Protocol Registration Status

Item	Details
Registration status	Registered in INPLASY (registration number: INPLASY202650156; doi: 10.37766/inplasy2026.5.0156; registration date: May 28, 2026; status: Published)
Reason	This review originated as a master's thesis submitted to Chungbuk National University. Prospective registration was not completed prior to search initiation. Registration in INPLASY (INPLASY202650156; doi: 10.37766/inplasy2026.5.0156) was subsequently completed on May 28, 2026, following manuscript preparation. Minor deviations from the registered protocol: (1) search extended to February 2025 (registered: December 2024); (2) age range 40–65 years used (registered: 40–64); (3) CMA v4.0 used (registered: v3.0). These deviations did not affect primary outcomes or conclusions.
Protocol finalized	March 1, 2025 (prior to search initiation)
Search conducted	March 1–7, 2025
Data extraction completed	March 2025
Analysis completed	April 2025
Transparency measures	Full coding sheet (Table S1), analysis scripts (Documents S5–S7), and this retrospective summary provided

2. Pre-Specified Eligibility Criteria (PICOTS-SD)

Finalized: March 1, 2025 (before search initiation)

Element	Inclusion Criteria	Exclusion Criteria
P (Population)	Women aged 40–65 years (middle-aged); Korean women (following systematic search outcome)	Men; mixed-gender without sex-stratified data; age outside 40–65
I (Intervention)	Forest therapy programs: instructor-led, researcher-designed, or indoor-based	Urban parks only; non-forest nature-based interventions; pharmacological interventions
C (Comparator)	Waitlist control, usual care, or active control (meta-analysis only)	No comparator (included in systematic review only)
O (Outcomes)	Psychological (stress, depression, anxiety, QoL), physiological (cortisol, BP, NK cells), physical (fitness, BMI)	Non-health outcomes
T (Timeframe)	No restriction; classified by session count, duration, format	—
S (Setting)	General population or	—

	symptomatic/disease groups	
SD (Study Design)	RCTs, NRCTs (meta-analysis); single-group pre-post designs (systematic review only)	Case reports, qualitative studies, narrative reviews

3. Pre-Specified Databases and Search Terms

Finalized: March 1, 2025

Database	Type	Search Period
RISS, DBpia, KISS, ScienceON, National Assembly Library	Domestic (Korean)	Jan 2000 – Feb 2025
Web of Science, Scopus, PubMed, MEDLINE, EMBASE	International	Jan 2000 – Feb 2025

4. Pre-Specified Statistical Analysis Decisions

Finalized: March 1, 2025 (prior to data extraction)

Decision	Specification
Primary effect size metric	Hedges' g (bias-corrected; Hedges & Olkin, 1985)
Primary analytical model	Three-level random-effects model with REML estimation and robust variance estimation (RVE, CR2 correction) using R packages metafor (v4.8-0) and robumeta (v2.1)
Supplementary model	DerSimonian–Laird random-effects model (CMA v4.0) for comparability with prior literature
Handling of multiple effect sizes	Multiple outcomes per study coded as independent effect sizes (k = 128); dependency addressed via three-level modeling
Moderator analyses	14 pre-specified categorical moderator variables (exploratory; no formal multiple-comparison correction)
Heterogeneity indices	Cochran's Q, I^2, tau, tau^2, 95% prediction interval
Publication bias	Egger's regression, Begg–Mazumdar rank correlation, Rosenthal fail-safe N, trim-and-fill, PET-PEESE, selection model
Certainty of evidence	GRADE framework (Guyatt et al., 2011)
Sensitivity analysis	Leave-one-out analysis (effect-level, k = 128); supplementary one-effect-per-study sensitivity check
Software	R version 4.4.3; CMA v4.0 (supplementary)
No post-hoc additions	No primary outcome or moderator variable was added following data extraction

5. Author Attestation

The authors attest that the eligibility criteria (PICOTS-SD), database selection, search terms, primary statistical approach, and all 14 moderator variables were determined prior to the formal data extraction phase. No substantive analytical decisions were made after reviewing the data.

Corresponding Author: Pyeong-Sik Yeon (well@chungbuk.ac.kr)

Date of attestation: May 28, 2026 | INPLASY Registration: INPLASY202650156 | doi: 10.37766/inplasy2026.5.0156
