# Supplementary material for: Validating the Effectiveness of Forest Therapy Programs for Middle-Aged Korean Women: A Systematic Review and Meta-Analytic Approach
Source: Healthcare (Basel). 2026 Jun 3;14(11):1569. doi: 10.3390/healthcare14111569 (PMC13257257; doi:10.3390/healthcare14111569)
Supplement: Supplementary file 1 [file healthcare-14-01569-s001.zip › Document_S2_analysis_3level.pdf]

Document S2: analysis_3level.R — Three-Level Random-Effects Meta-Analysis with RVE (CR2)

Manuscript: Validating the Effectiveness of Forest Therapy Programs for Middle-Aged Women: A Systematic Review and Meta-Analytic Approach
Journal: *Healthcare* (MDPI) | R version: 4.4.3 | Packages: metafor 4.8-0, robumeta 2.1

```r
# ============================================================
# Document S5: analysis_3level.R
# Three-Level Random-Effects Meta-Analysis with RVE (CR2)
#
# Manuscript: Validating the Effectiveness of Forest Therapy
# Programs for Middle-Aged Women: A Systematic Review and
# Meta-Analytic Approach
# Journal: Healthcare (MDPI)
# Authors: Young-Ho Lee, Gyeong-Min Min, Pyeong-Sik Yeon
#
# R version: 4.4.3
# Required packages: metafor (>= 4.8-0), robumeta (>= 2.1)
# Run AFTER compute_effects.R
# ============================================================

# ------ 1. Setup --------------------------------------------
library(metafor)
library(robumeta)

# Load analytic dataset (output of compute_effects.R)
dat <- read.csv("analytic_data.csv")
cat("Data loaded: k =", nrow(dat), "| Studies =",
length(unique(dat$study_group)), "\n\n")

# ------ 2. PRIMARY ANALYSIS ---------------------------------
# Three-level random-effects model
# Level 1: sampling variance (known, = vi)
# Level 2: within-study variance (effects nested within studies)
# Level 3: between-study variance
#
# Estimated via REML; robust variance estimation (CR2) applied
# to correct for potential model misspecification.
# ----------------------------------------------------------

mod3 <- rma.mv(
yi = yi,
V = vi,
random = ~ 1 | study_group / effect_id, # proper nesting: effects within studies
method = "REML",
data = dat,
digits = 6
)

cat("=== THREE-LEVEL MODEL SUMMARY ===\n")
print(summary(mod3))

# Extract variance components
tau2_between <- mod3$sigma2[1] # between-study (Level 3)
tau2_within <- mod3$sigma2[2] # within-study (Level 2)
```

```r
cat("\n--- Variance Components ---\n")
cat("tau2 between-study (sigma^2.1):", round(tau2_between, 6), "\n")
cat("tau2 within-study (sigma^2.2):", round(tau2_within, 6), "\n")
cat("tau2 total:", round(tau2_between + tau2_within, 6), "\n")

# I-squared decomposition
W <- diag(1 / dat$vi)
X <- model.matrix(mod3)
P <- W - W %*% X %*% solve(t(X) %*% W %*% X) %*% t(X) %*% W
I2_total <- 100 * sum(mod3$sigma2) / (sum(mod3$sigma2) + (mod3$k - mod3$p) / sum(diag(P)))
I2_between <- 100 * tau2_between / (sum(mod3$sigma2) + (mod3$k - mod3$p) / sum(diag(P)))
I2_within <- 100 * tau2_within / (sum(mod3$sigma2) + (mod3$k - mod3$p) / sum(diag(P)))

cat("\n--- Heterogeneity (I² decomposition) ---\n")
cat("I² total :", round(I2_total, 1), "%\n")
cat("I² between :", round(I2_between, 1), "%\n")
cat("I² within :", round(I2_within, 1), "%\n")

# ------ 3. ROBUST VARIANCE ESTIMATION (CR2) -----------------
# Applies CR2 small-sample correction for heteroscedasticity
# Reference: Pustejovsky & Tipton (2018); clubSandwich package
# robumeta provides a simplified RVE implementation.

rve_fit <- robu(
formula = yi ~ 1,
data = dat,
studynum = study_group,
var.eff.size = vi,
modelweights = "CORR",
small = TRUE # CR2 correction
)

cat("\n=== ROBUST VARIANCE ESTIMATION (CR2) ===\n")
print(summary(rve_fit))

# Primary reported result
g_primary <- rve_fit$reg_table$b.r[1]
se_primary <- rve_fit$reg_table$SE[1]
cat("\n--- PRIMARY RESULT (RVE CR2) ---\n")
cat("Pooled Hedges' g:", round(g_primary, 4), "\n")
cat("SE: ", round(se_primary, 4), "\n")
cat("95% CI: ", round(g_primary - 1.96*se_primary, 4),
"to", round(g_primary + 1.96*se_primary, 4), "\n")

# ------ 4. PREDICTION INTERVAL ------------------------------
PI_lo <- mod3$b[1] - qnorm(0.975) * sqrt(sum(mod3$sigma2) + mod3$se^2)
PI_hi <- mod3$b[1] + qnorm(0.975) * sqrt(sum(mod3$sigma2) + mod3$se^2)
cat("\n--- 95% Prediction Interval ---\n")
cat("PI: [", round(PI_lo, 4), ",", round(PI_hi, 4), "]\n")
```

```r
# ------ 5. SUPPLEMENTARY: DL RANDOM-EFFECTS MODEL ----------
# Standard DerSimonian-Laird model for literature comparability
# (treats all 128 effects as independent — reported as supplementary)
mod_dl <- rma(yi = yi, vi = vi, method = "DL", data = dat)
cat("\n=== SUPPLEMENTARY: DERSIMONIAN-LAIRD MODEL ===\n")
cat("Pooled g:", round(mod_dl$b[1], 4),
"| 95% CI:", round(mod_dl$ci.lb, 4), "to", round(mod_dl$ci.ub, 4), "\n")
cat("I²:", round(mod_dl$I2, 1), "%",
"| Q(df=127):", round(mod_dl$QE, 2), ", p <", format.pval(mod_dl$QEp, eps=0.001), "\n")

# ------ 6. SAVE KEY RESULTS ---------------------------------
results <- list(
primary_g = round(g_primary, 4),
primary_se = round(se_primary, 4),
tau2_between = round(tau2_between, 4),
tau2_within = round(tau2_within, 4),
I2_total = round(I2_total, 1),
I2_between = round(I2_between, 1),
I2_within = round(I2_within, 1),
PI_lo = round(PI_lo, 4),
PI_hi = round(PI_hi, 4),
DL_g = round(mod_dl$b[1], 4),
DL_CI_lo = round(mod_dl$ci.lb, 4),
DL_CI_hi = round(mod_dl$ci.ub, 4),
DL_I2 = round(mod_dl$I2, 1),
DL_Q = round(mod_dl$QE, 2)
)

write.csv(as.data.frame(results), "mod3_stats_output.csv", row.names = FALSE)
saveRDS(mod3, "mod3_model.rds")

cat("\nResults saved: mod3_stats_output.csv, mod3_model.rds\n")
cat("\nExpected primary result: g ≈ 0.596, SE ≈ 0.081 (manuscript Table 2)\n")

# ==============================================================
# END OF analysis_3level.R
# Next step: run analysis_RVE_sensitivity.R
# ==============================================================
```
