# Supplementary material for: Validating the Effectiveness of Forest Therapy Programs for Middle-Aged Korean Women: A Systematic Review and Meta-Analytic Approach
Source: Healthcare (Basel). 2026 Jun 3;14(11):1569. doi: 10.3390/healthcare14111569 (PMC13257257; doi:10.3390/healthcare14111569)
Supplement: Supplementary file 1 [file healthcare-14-01569-s001.zip › Document_S3_compute_effects.pdf]

Document S3: compute_effects.R — Effect Size Computation (Hedges' g)

Manuscript: Validating the Effectiveness of Forest Therapy Programs for Middle-Aged Women: A Systematic Review and Meta-Analytic Approach
Journal: *Healthcare* (MDPI) | R version: 4.4.3 | Packages: metafor 4.8-0, robumeta 2.1

```
# ============================================================
# Document S6: compute_effects.R
# Effect Size Computation (Hedges' g) from Raw Group Statistics
#
# Manuscript: Validating the Effectiveness of Forest Therapy
# Programs for Middle-Aged Women: A Systematic Review and
# Meta-Analytic Approach
# Journal: Healthcare (MDPI)
# Authors: Young-Ho Lee, Gyeong-Min Min, Pyeong-Sik Yeon
#
# R version: 4.4.3
# Required packages: metafor (>= 4.8-0)
# Run this script FIRST (before analysis_3level.R)
# ============================================================

# ------ 1. Setup ---------------------------------------------
if (!requireNamespace("metafor", quietly = TRUE))
install.packages("metafor")
library(metafor)

# ------ 2. Load raw data ------------------------------------
raw <- read.csv("Table_S1_Effect_Level_Data.xlsx",
na.strings = c("", "NA"))
# If loading from CSV:
# raw <- read.csv("effect_level_data_filled.csv", na.strings = c("", "NA"))

# Required columns:
# n1 = experimental group n
# n2 = control group n
# Exp.Mean = experimental group mean
# Exp.SD = experimental group SD
# Control.Mean= control group mean
# Control.SD = control group SD
# effect_id = unique effect size ID (1-128)
# study_group = actual study identifier (1-24; see below)

# ------ 3. Define study_group (24 parent studies) -----------
# Maps effect_id ranges to the 24 actual studies.
# effect_id ranges per study (from Table A1 in manuscript):
study_ranges <- data.frame(
study_group = 1:24,
author = c("Kim_AR","Kim_HG","Kim_HS","Min_JW","Park_BJ",
"Park_JS","Park_CE","Shin_DJ","Shin_MJ","Oh_JY",
"Yoo_SR","Yoon_MA","Lee_MO","Lee_YJ","Lee_JS",
"Jeong_SR","Jeong_YG","Jeong_HJ","Choi_G","Choi_JH_2010",
"Choi_JH_2018","Choi_JH_2016a","Choi_JH_2016b","Hong_JS"),
year = c(2024,2016,2018,2023,2022,2023,2019,2023,2016,2015,
2015,2020,2018,2020,2016,2019,2023,2023,2023,2010,
2018,2016,2016,2019),
eid_start = c(1,5,7,10,15,21,26,50,52,55,
```

```
58,66,67,75,86,95,99,102,105,108,
111,118,123,125),
eid_end = c(4,6,9,14,20,25,49,51,54,57,
65,66,74,85,94,98,101,104,107,110,
117,122,124,128)
)

# Assign study_group to each effect
raw$study_group <- NA
for (i in seq_len(nrow(study_ranges))) {
idx <- raw$effect_id >= study_ranges$eid_start[i] &
raw$effect_id <= study_ranges$eid_end[i]
raw$study_group[idx] <- study_ranges$study_group[i]
}

cat("study_group assignment check:\n")
print(table(raw$study_group))
cat("Total effects:", nrow(raw), "\n")
cat("Unique studies:", length(unique(raw$study_group)), "\n\n")

# ------ 4. Compute Hedges' g using escalc() -----------------
# Method: SMD (standardized mean difference) with Hedges' correction
# Formula: (M_exp - M_ctrl) / pooled_SD * correction_factor
#
# Column mapping (adjust if your column names differ):
dat <- escalc(
measure = "SMD", # Standardized Mean Difference = Hedges' g
m1i = raw$Experimental..n., # experimental mean → NOTE: check column names
sd1i = raw$Experimental.SD, # experimental SD
n1i = raw$n1, # experimental n
m2i = raw$Control.Mean, # control mean
sd2i = raw$Control.SD, # control SD
n2i = raw$n2, # control n
data = raw,
var.names = c("yi_computed", "vi_computed"),
append = TRUE
)

# Note: escalc SMD applies Hedges' small-sample correction automatically.

# ------ 5. Validate against pre-computed values -------------
cat("Validation: computed vs. pre-loaded yi\n")
cat("Correlation:", round(cor(dat$yi_computed, dat$yi, use = "complete.obs"), 6), "\n")
cat("Max absolute difference:",
round(max(abs(dat$yi_computed - dat$yi), na.rm = TRUE), 6), "\n\n")

# ------ 6. Use pre-computed yi/vi (from Table S1) -----------
# The manuscript uses yi and vi as already stored in the coding sheet.
# These are verified via the correlation check above (r ≈ 1.00).
dat$study_group <- raw$study_group
```

```
# ------ 7. Save final analytic dataset -----------------------
write.csv(dat[, c("effect_id","study_group","yi","vi",
"Outcome.Category","Study.Design..RCT.NRCT.Pre.Post.",
"Program.Format..Same.day.Residential.",
"Setting","Intervention.Content",
"Total.Duration..Weeks.","n1","n2")],
"analytic_data.csv", row.names = FALSE)

cat("Analytic dataset saved: analytic_data.csv\n")
cat("k =", nrow(dat), "| Studies =", length(unique(dat$study_group)), "\n")
cat("yi range:", round(min(dat$yi), 3), "to", round(max(dat$yi), 3), "\n")
cat("vi range:", round(min(dat$vi), 5), "to", round(max(dat$vi), 5), "\n")

# ============================================================
# END OF compute_effects.R
# Next step: run analysis_3level.R
# ============================================================
```
