# Supplementary material for: Validating the Effectiveness of Forest Therapy Programs for Middle-Aged Korean Women: A Systematic Review and Meta-Analytic Approach
Source: Healthcare (Basel). 2026 Jun 3;14(11):1569. doi: 10.3390/healthcare14111569 (PMC13257257; doi:10.3390/healthcare14111569)
Supplement: Supplementary file 1 [file healthcare-14-01569-s001.zip › Document_S4_analysis_RVE_sensitivity.pdf]

Document S4: analysis_RVE_sensitivity.R — Sensitivity Analyses: Leave-One-Out, Publication Bias

Manuscript: Validating the Effectiveness of Forest Therapy Programs for Middle-Aged Women: A Systematic Review and Meta-Analytic Approach
Journal: *Healthcare* (MDPI) | R version: 4.4.3 | Packages: metafor 4.8-0, robumeta 2.1

```
# ===============================================================
# Document S7: analysis_RVE_sensitivity.R
# Sensitivity Analyses: Leave-One-Out, Publication Bias,
# PET-PEESE, Selection Model
#
# Manuscript: Validating the Effectiveness of Forest Therapy
# Programs for Middle-Aged Women: A Systematic Review and
# Meta-Analytic Approach
# Journal: Healthcare (MDPI)
# Authors: Young-Ho Lee, Gyeong-Min Min, Pyeong-Sik Yeon
#
# R version: 4.4.3
# Required packages: metafor (>= 4.8-0), robumeta (>= 2.1)
# Run AFTER analysis_3level.R
# ===============================================================

# ------ 1. Setup ---------------------------------------------
library(metafor)
library(robumeta)

dat <- read.csv("analytic_data.csv")
mod3 <- readRDS("mod3_model.rds")

cat("Data loaded: k =", nrow(dat), "| Studies =",
length(unique(dat$study_group)), "\n\n")

# ------ 2. LEAVE-ONE-OUT SENSITIVITY (effect-level) ---------
# Removes one effect size at a time and re-estimates pooled g
# Tests whether any single effect drives the overall conclusion

cat("=== LEAVE-ONE-OUT ANALYSIS (k = 128) ===\n")
loo_results <- data.frame(
effect_id = dat$effect_id,
study_group = dat$study_group,
pooled_g = NA_real_,
se = NA_real_,
ci_lo = NA_real_,
ci_hi = NA_real_,
delta_g = NA_real_
)

for (i in seq_len(nrow(dat))) {
dat_sub <- dat[-i, ]
tryCatch({
m_sub <- rma.mv(
yi = yi, V = vi,
random = ~ 1 | study_group / effect_id,
method = "REML", data = dat_sub
)
rve_sub <- robu(
```

```
formula = yi ~ 1, data = dat_sub,
studynum = study_group, var.eff.size = vi,
modelweights = "CORR", small = TRUE
)
g_i <- rve_sub$reg_table$b.r[1]
se_i <- rve_sub$reg_table$SE[1]
loo_results$pooled_g[i] <- round(g_i, 6)
loo_results$se[i] <- round(se_i, 6)
loo_results$ci_lo[i] <- round(g_i - 1.96 * se_i, 6)
loo_results$ci_hi[i] <- round(g_i + 1.96 * se_i, 6)
}, error = function(e) {
cat("Error at effect_id", dat$effect_id[i], ":", conditionMessage(e), "\n")
})
if (i %% 20 == 0) cat(" Completed:", i, "/ 128\n")
}

# Original g
g_orig <- 0.5959 # from primary analysis
loo_results$delta_g <- round(loo_results$pooled_g - g_orig, 6)
loo_results$abs_delta_g <- abs(loo_results$delta_g)
loo_results$influence <- ifelse(loo_results$abs_delta_g >= 0.05, "High",
ifelse(loo_results$abs_delta_g >= 0.02, "Moderate", "Minimal"))

cat("\n--- Leave-One-Out Summary ---\n")
cat("Original g:", g_orig, "\n")
cat("Range after removal:", round(min(loo_results$pooled_g, na.rm=T), 4),
"to", round(max(loo_results$pooled_g, na.rm=T), 4), "\n")
cat("High influence effects (|Δg| ≥ 0.05):",
sum(loo_results$influence == "High", na.rm=T), "\n")
cat("Moderate influence (0.02 ≤ |Δg| < 0.05):",
sum(loo_results$influence == "Moderate", na.rm=T), "\n")
cat("Minimal influence (|Δg| < 0.02):",
sum(loo_results$influence == "Minimal", na.rm=T), "\n")

write.csv(loo_results, "leave_one_out_results_v2.csv", row.names = FALSE)
cat("Saved: leave_one_out_results_v2.csv\n\n")

# ------ 3. PUBLICATION BIAS DIAGNOSTICS ---------------------
# Using standard DL model for bias tests (k=128 independent effects)
mod_dl <- rma(yi = yi, vi = vi, method = "DL", data = dat)

cat("=== PUBLICATION BIAS DIAGNOSTICS ===\n")

# 3a. Funnel plot
pdf("funnel_plot.pdf", width=7, height=6)
funnel(mod_dl,
xlab = "Hedges' g",
main = "Funnel Plot (Standard Error)",
pch = 19, col = rgb(0.2, 0.4, 0.8, 0.6))
abline(v = mod_dl$b[1], lty = 2, col = "red")
```

```
dev.off()
cat("Funnel plot saved: funnel_plot.pdf\n")

# 3b. Egger's regression test
egger <- regtest(mod_dl, model = "rma", predictor = "sei")
cat("\nEgger's Regression Test:\n")
cat(" t =", round(egger$zval, 4), ", p =", round(egger$pval, 4), "\n")

# 3c. Begg-Mazumdar rank correlation
begg <- ranktest(mod_dl)
cat("\nBegg-Mazumdar Rank Correlation:\n")
cat(" tau =", round(begg$tau, 4), ", p =", round(begg$pval, 4), "\n")

# 3d. Trim-and-Fill
tf <- trimfill(mod_dl)
cat("\nTrim-and-Fill:\n")
cat(" Imputed studies:", tf$k0, "\n")
if (tf$k0 > 0) cat(" Adjusted g:", round(tf$b[1], 4), "\n")

# 3e. Rosenthal Fail-Safe N
fsn_result <- fsn(yi = yi, vi = vi, data = dat, type = "Rosenthal")
cat("\nRosenthal Fail-Safe N:\n")
cat(" N =", fsn_result$fsnum, "\n")
cat(" Tolerance threshold (5k+10):", 5*nrow(dat)+10, "\n")

# ------ 4. PET-PEESE PUBLICATION BIAS CORRECTION -----------
# PET: Precision-Effect Test
# PEESE: Precision-Effect Estimate with Standard Error
# Reference: Stanley & Doucouliagos (2014)

cat("\n=== PET-PEESE ANALYSIS ===\n")

# PET: regress yi on SE (standard error)
dat$sei <- sqrt(dat$vi)
pet_mod <- rma(yi ~ sei, vi = vi, method = "DL", data = dat)
cat("PET (yi ~ SE):\n")
cat(" Intercept (bias-corrected g):", round(pet_mod$b[1], 4),
"p =", round(pet_mod$pval[1], 4), "\n")
cat(" SE coefficient:", round(pet_mod$b[2], 4),
"p =", round(pet_mod$pval[2], 4), "\n")

# PEESE: regress yi on vi (variance)
peese_mod <- rma(yi ~ vi, vi = vi, method = "DL", data = dat)
cat("\nPEESE (yi ~ vi):\n")
cat(" Intercept (bias-corrected g):", round(peese_mod$b[1], 4),
"p =", round(peese_mod$pval[1], 4), "\n")
cat(" vi coefficient:", round(peese_mod$b[2], 4),
"p =", round(peese_mod$pval[2], 4), "\n")

# Decision rule: if PET intercept significant → use PEESE
```

```
if (pet_mod$pval[1] < 0.05) {
cat("\nDecision: PET intercept significant → report PEESE estimate\n")
cat("Bias-adjusted pooled g (PEESE):", round(peese_mod$b[1], 4), "\n")
} else {
cat("\nDecision: PET intercept non-significant → report PET estimate\n")
cat("Bias-adjusted pooled g (PET):", round(peese_mod$b[1], 4), "\n")
}

# ------ 5. SELECTION MODEL (3PSM) ---------------------------
# Three-parameter selection model
# Reference: Vevea & Hedges (1995) via metafor
cat("\n=== SELECTION MODEL (3PSM) ===\n")
tryCatch({
sel_mod <- selmodel(mod_dl, type = "stepfun",
steps = 0.025,
delta = 1)
cat("Selection model (p < 0.025 threshold):\n")
cat(" Adjusted g:", round(coef(sel_mod)[1], 4), "\n")
print(summary(sel_mod))
}, error = function(e) {
cat("Selection model not converged:", conditionMessage(e), "\n")
cat("Interpretation: high heterogeneity (I² = 75.1%) prevents stable estimation.\n")
})

# ------ 6. SENSITIVITY: ONE EFFECT PER STUDY ---------------
# Aggregate to study-level (representative effect per study)
# as supplementary check for dependency
cat("\n=== SENSITIVITY: ONE EFFECT PER STUDY (n = 24) ===\n")
dat_agg <- aggregate(
cbind(yi, vi) ~ study_group,
data = dat,
FUN = function(x) mean(x, na.rm = TRUE)
)
dat_agg <- dat_agg[order(dat_agg$study_group), ]

mod_agg <- rma(yi = yi, vi = vi, method = "REML", data = dat_agg)
cat("Study-level aggregated model (k = 24):\n")
cat(" g =", round(mod_agg$b[1], 4),
"| 95% CI:", round(mod_agg$ci.lb, 4), "to", round(mod_agg$ci.ub, 4), "\n")
cat(" I² =", round(mod_agg$I2, 1), "%\n")

# ------ 7. SAVE SUMMARY TABLE --------------------------------
pub_bias_summary <- data.frame(
Test = c("Egger's regression", "Begg-Mazumdar",
"Trim-and-fill (imputed k)", "Rosenthal FSN",
"PET intercept", "PEESE intercept",
"Study-level agg. g (k=24)"),
Statistic = c(round(egger$zval, 3), round(begg$tau, 3),
tf$k0, fsn_result$fsnum,
round(pet_mod$b[1], 4), round(peese_mod$b[1], 4),
```

```
round(mod_agg$b[1], 4)),
p_value = c(round(egger$pval, 4), round(begg$pval, 4),
NA, NA,
round(pet_mod$pval[1], 4), round(peese_mod$pval[1], 4),
round(mod_agg$pval, 4)),
Interpretation = c(
ifelse(egger$pval < 0.05, "Asymmetry indicated", "No asymmetry"),
ifelse(begg$pval < 0.05, "Asymmetry indicated", "No asymmetry"),
ifelse(tf$k0 == 0, "No imputation needed", paste(tf$k0, "studies imputed")),
paste("Exceeds threshold (", 5*nrow(dat)+10, ")"),
ifelse(pet_mod$pval[1] < 0.05, "Bias present; use PEESE", "No significant bias"),
paste("Bias-adjusted estimate:", round(peese_mod$b[1], 4)),
"Consistent with primary result"
)
)

write.csv(pub_bias_summary, "publication_bias_summary.csv", row.names = FALSE)
cat("\nSaved: publication_bias_summary.csv\n")

cat("\n========================================\n")
cat("All sensitivity analyses complete.\n")
cat("Key files saved:\n")
cat(" leave_one_out_results_v2.csv\n")
cat(" publication_bias_summary.csv\n")
cat(" funnel_plot.pdf\n")
cat("========================================\n")

# =============================================================
# END OF analysis_RVE_sensitivity.R
# =============================================================
```
