# Supplementary material for: Validating the Effectiveness of Forest Therapy Programs for Middle-Aged Korean Women: A Systematic Review and Meta-Analytic Approach
Source: Healthcare (Basel). 2026 Jun 3;14(11):1569. doi: 10.3390/healthcare14111569 (PMC13257257; doi:10.3390/healthcare14111569)
Supplement: Supplementary file 1 [file healthcare-14-01569-s001.zip › Table_S1_Effect_Level_Data.pdf]

Table S1. Effect-Level Data Extraction and Coding Sheet (k = 128 Effect Sizes, 24 Studies)

k = 128 effect sizes | 24 studies | Journal: Healthcare (MDPI)

Study No.	First Author	Year	Study Design	Participant Characteristics	Age Range	Setting	Intervention Type	Intervention Content	Control Program	Outcome Domain	Program Format	Residential Nights	Session Format	Total Duration (Weeks)	Sessions/ Week	Total Sessions	Session Duration	Outcome Category	Outcome Measure	Control n	Control Mean	Control SD	Exp n	Exp Mean	Exp SD	Mean Diff	Pooled SD	Hedges' g	yi	vi	effect_id	study_id
1	Kim, A.R.	2024	NRCT	General Women	40–59 yrs	Forest Bathing Site	Unstructured	Expressive writing		Psychological	Same-day /Multi-session		Multi-session	6 wks	2x/wk	10 sessions	≤2 hrs	Psychological	Perceived Stress	12	-1.63	0.3500	12	-1.7000	0.5500	-0.0700	0.4600	-0.1500	-0.1500	0.1558	1	1
1	Kim, A.R.	2024	NRCT	General Women															Parenting Stress	12	-1.84	0.0800	12	-1.7500	0.8700	0.0900	0.6100	0.1500	0.1500	0.1558	2	2
1	Kim, A.R.	2024	NRCT	General Women															Depression (longitudinal)	12	-0.78	0.4000	12	-0.5800	0.2700	0.2000	0.3500	0.5800	0.5800	0.1619	3	3
1	Kim, A.R.	2024	NRCT	General Women															Somatization (longitudinal)	12	-0.85	0.5600	12	-0.3700	0.4100	0.4800	0.5000	0.9600	0.9600	0.1733	4	4
2	Kim, H.G.	2016	RCT	General Women	55±2.01 yrs	Healing Forest	Structured	EFT (10 min) + Forest meditation walking (60 min)	1. Forest meditation walking_x000D_ 2. Forest EFT_x000D_ 3. Urban EFT (three conditions)	Psychological	Same-day /Multi-session		Multi-session	6 wks	1x/wk	10 sessions	≤2 hrs	Psychological	Climacteric Symptoms (MENSI; Menopause Symptom Index)	6	-17.13	6.2700	8	-9.1300	6.6900	8	6.4200	1.2500	1.2500	0.3045	5	5
2	Kim, H.G.	2016	RCT																Quality of Life (WHOQOL-BREF)	6	91.67	12.5600	8	97.3800	12.2600	5.7100	12.4100	0.4600	0.4600	0.2622	6	6
3	Kim, H.S.	2018	NRCT	General Women	55.4 (±2.0) yrs	Healing Forest	Structured	Walking, meditation, gymnastics	Forest gymnastics + forest trail walk (n=7), forest gymnastics only (n=7), forest meditation (n=7), indoor urban activities (n=7)	Psychological/Physiological	Same-day /Multi-session		Multi-session	4 wks	1x/wk	10 sessions	≤2 hrs	Psychological	Depression	7	-56.8	4.3800	7	-41.1000	5.0100	15.7000	4.6600	3.3700	3.3700	0.6059	7	7
3	Kim, H.S.	2018	NRCT																Stress	7	-65.4	6.9800	7	-59	5.0300	6.4000	6.1300	1.0400	1.0400	0.2843	8	8
3	Kim, H.S.	2018	NRCT																Quality of Life	7	70.0	6.1600	7	80.6000	9.3600	10.6000	8.0300	1.3200	1.3200	0.3049	9	9
4	Min, J.W.	2023	RCT	General Women	54.25±3.11	Indoor	Unstructured	Stretching, 4 resistance exercises, 4 aerobic exercises	Phytoncide diffusion group / Non-diffusion group	Physiological	Same-day /Multi-session		Multi-session	12 wks	3x/wk	40 sessions	≤2 hrs	Physiological	Blood Lactate	12	-3.96	0.3900	12	-3.6500	0.6000	0.3100	0.5000	0.6200	0.6200	0.1628	10	10
4	Min, J.W.	2023	RCT																Blood Triglyceride	12	-159.50	5.3700	12	-155.9200	7.3800	3.5800	6.4400	0.5600	0.5600	0.1615	11	11
4	Min, J.W.	2023	RCT																Stress Index	12	-14.66	2.6300	12	-13.1800	3.1600	1.4800	2.8900	0.5100	0.5100	0.1604	12	12
4	Min, J.W.	2023	RCT																EEG Index	12	5.10	1.1300	12	4.9100	0.5900	-0.1900	0.9100	-0.2100	-0.2100	0.1562	13	13
4	Min, J.W.	2023	RCT																Antioxidant Index	12	2.03	0.2900	12	1.5700	0.7300	-0.4600	0.4900	-0.9300	-0.9300	0.1722	14	14
5	Park, B.J.	2022	RCT	General Women	Mean 53 yrs	Healing Forest	Unstructured	Meditation-centered forest therapy program	Group A: forest (3 days) → urban (3 days); Group B: urban (3 days) → forest (3 days)	Psychological	Residential	2 nights/3 days	Single-session	2 wks	3x/wk	10 sessions	≤2 hrs	Psychological	Tension	53	-2.25	3.1100	53	-1.7800	2.5500	0.4700	2.8700	0.1600	0.1600	0.0373	15	15
5	Park, B.J.	2022	RCT																Depression	53	-2.15	3.5100	53	-1.7800	2.5500	0.3700	3.1000	0.1200	0.1200	0.0373	16	16
5	Park, B.J.	2022	RCT																Anger	53	-1.90	3.2000	53	-1.3300	2.1200	0.5700	2.7700	0.2100	0.2100	0.0374	17	17
5	Park, B.J.	2022	RCT																Vigor	53	9.40	5.1100	53	10.0800	6.3600	0.6800	5.7900	0.1200	0.1200	0.0373	18	18
5	Park, B.J.	2022	RCT																Fatigue	53	-3.05	3.3500	53	-1.8000	2.4600	1.2500	3.0200	0.4100	0.4100	0.0380	19	19
5	Park, B.J.	2022	RCT																Confusion	53	-4.18	2.5400	53	-3.8300	1.6500	0.3500	2.1600	0.1600	0.1600	0.0373	20	20
6	Park, J.S.	2023	NRCT	Breast Cancer Patients	50.33±7.53	Forest Trail	Unstructured	Band exercise, aerobic hiking	6 breast cancer survivors / 6 general middle-aged women	Physiological/Physical	Same-day /Multi-session		Multi-session	2 wks	2x/wk	20 sessions	≤2 hrs	Physiological	Cortisol	6	-9.53	2.3200	6	-12.2000	6.4900	-2.6700	5.0800	-0.5300	-0.5300	0.2940	21	21
6	Park, J.S.	2023	NRCT																Isokinetic Muscle Function (60° Knee Extension)	6	54.40	3.5900	6	64.8200	7.2000	10.4200	5.8400	1.7800	1.7800	0.3965	22	22
6	Park, J.S.	2023	NRCT																60° Knee Flexion	6	47.40	1.4400	6	50.8500	4.3000	3.4500	3.2500	1.0600	1.0600	0.3239	23	23
6	Park, J.S.	2023	NRCT																Dynamic Balance (Left)	6	98.20	2.2500	6	98.9400	4.7500	0.7400	3.7200	0.2000	0.2000	0.2854	24	24

Study No.	First Author	Year	Study Design	Participant Characteristics	Age Range	Setting	Intervention Type	Intervention Content	Control Program	Outcome Domain	Program Format	Residential Nights	Session Format	Total Duration (Weeks)	Sessions/ Week	Total Sessions	Session Duration	Outcome Category	Outcome Measure	Control n	Control Mean	Control SD	Exp n	Exp Mean	Exp SD	Mean Diff	Pooled SD	Hedges' g	yi	vi	effect_id	study_id
6	Park, J.S.	2023	NRCT																Dynamic Balance (Right)	6	101.08	3.8100	6	98.0800	5.7300	-3	4.8500	-0.6200	-0.6200	0.2977	25	25
7	Park, C.E.	2019	RCT	General Women	40–65 yrs (Mean 53)	Healing Forest	Structured	Supine meditation, standing yoga, walking, healing touch, supine meditation, stress management, nature meditation	Experimental group (urban then forest) / Control group (forest then urban)	Psychological/Physiological	Residential	2 nights/3 days	Single-session	2 wks	1x/wk	10 sessions	≤8 hrs	Physiological	Serotonin	53	134.22	53.4000	53	156.2800	57.8300	22.0600	55.8300	0.4000	0.4000	0.0379	26	26
7	Park, C.E.	2019	RCT																Vitamin D	53	16.95	7.0200	53	18.1100	7.8400	1.1600	7.4600	0.1600	0.1600	0.0373	27	27
7	Park, C.E.	2019	RCT																HRV (HF Component)	53	171.41	130.9100	53	122.8100	115.2500	-48.6000	124.6200	-0.3900	-0.3900	0.0379	28	28
7	Park, C.E.	2019	RCT																HRV (LF/HF Ratio)	53	4.10	6.3700	53	7.1800	11.1600	3.0800	9.8200	0.3100	0.3100	0.0376	29	29
7	Park, C.E.	2019	RCT																Socio-psychological Stress	40	-15.15	8.2300	40	-14.3800	8.8300	0.7700	8.5300	0.0900	0.0900	0.0491	30	30
7	Park, C.E.	2019	RCT																Stress Response	40	-17.70	18.4400	40	-15.2500	16.4500	2.4500	17.5400	0.1400	0.1400	0.0492	31	31
7	Park, C.E.	2019	RCT																Tension	40	-2.88	3.5900	40	-2.3800	3.3700	0.5000	3.4900	0.1400	0.1400	0.0492	32	32
7	Park, C.E.	2019	RCT																Aggression	40	-0.33	0.6600	40	-0.2000	0.4100	0.1300	0.5600	0.2300	0.2300	0.0494	33	33
7	Park, C.E.	2019	RCT																Somatization	40	-1.35	1.6100	40	-1.2000	1.6400	0.1500	1.6200	0.0900	0.0900	0.0491	34	34
7	Park, C.E.	2019	RCT																Anger	40	-2.98	3.9000	40	-2.5800	3.1700	0.4000	3.5700	0.1100	0.1100	0.0491	35	35
7	Park, C.E.	2019	RCT																Depression	40	-3.15	3.8700	40	-3	3.9600	0.1500	3.9100	0.0400	0.0400	0.0490	36	36
7	Park, C.E.	2019	RCT																Fatigue	40	-3.93	3.0800	40	-3.2500	2.5200	0.6800	2.8300	0.2400	0.2400	0.0494	37	37
7	Park, C.E.	2019	RCT																Frustration	40	-3.10	4.3100	40	-2.6500	4.1900	0.4500	4.2500	0.1100	0.1100	0.0491	38	38
7	Park, C.E.	2019	RCT																Emotional Response	40	-5.08	5.5400	40	-3.8000	4.7900	1.2800	5.2200	0.2500	0.2500	0.0494	39	39
7	Park, C.E.	2019	RCT																Cognitive Response	40	6.58	6.8000	40	6.1000	6.4600	-0.4800	6.6400	-0.0700	-0.0700	0.0491	40	40
7	Park, C.E.	2019	RCT																Behavioral Response	40	-6.58	6.8000	40	-1.8300	2.2600	4.7500	5.9200	0.8000	0.8000	0.0530	41	41
7	Park, C.E.	2019	RCT																Physical Response	40	-3.78	3.3500	40	-3.5300	4.3700	0.2500	3.8800	0.0600	0.0600	0.0491	42	42
7	Park, C.E.	2019	RCT																Tension	40	-2.25	3.1100	40	-1.7800	2.5500	0.4700	2.8700	0.1600	0.1600	0.0492	43	43
7	Park, C.E.	2019	RCT																Depression	40	-2.15	3.5100	40	-1.7800	2.5500	0.3700	3.1000	0.1200	0.1200	0.0491	44	44
7	Park, C.E.	2019	RCT																Anger	40	-1.90	3.2000	40	-1.3300	2.1200	0.5700	2.7700	0.2100	0.2100	0.0493	45	45
7	Park, C.E.	2019	RCT																Vigor	40	9.40	5.1100	40	10.0800	6.3600	0.6800	5.7900	0.1200	0.1200	0.0491	46	46
7	Park, C.E.	2019	RCT																Fatigue	40	-3.05	3.3500	40	-1.8000	2.4600	1.2500	3.0200	0.4100	0.4100	0.0501	47	47
7	Park, C.E.	2019	RCT																Confusion	40	-4.18	2.5400	40	-3.8300	1.6500	0.3500	2.1600	0.1600	0.1600	0.0492	48	48
7	Park, C.E.	2019	RCT																Total Mood Disturbance (TMD)	40	- 4.13	17.0500	40	-0.0500	13.7100	4.0800	16.5300	0.2500	0.2500	0.0494	49	49
8	Shin, D.J.	2023	NRCT	General Women	40–65 yrs	Indoor	Unstructured	3 inhalation groups (hinoki cypress EO, lavender EO, mixed EO) + 1 non-inhalation group	160 participants assigned to 4 groups: hinoki cypress EO, lavender EO, mixed EO (inhalation groups), and non-inhalation group (control)	Psychological	Same-day/Multi-session		Multi-session	2 wks	7x/wk	20 sessions	≤4 hrs	Psychological	State Anxiety (K-STAI; Korean State-Trait Anxiety Inventory)	34	-41.12	7.5000	37	-37.0800	9.2000	4.0400	8.3500	0.4800	0.4800	0.0568	50	50
8	Shin, D.J.	2023	NRCT																Depression (CES-D; Center for Epidemiologic Studies Depression Scale)	34	-13.35	8.6900	37	-9.3800	7.4000	3.9700	8.1700	0.4900	0.4900	0.0569	51	51
9	Shin, M.J.	2016	RCT	General Women	50–59 yrs	Forest Trail	Unstructured	Walking exercise	Type I group (flat ground, low phytoncide; n=10); 60% HRR	Physiological	Same-day/Multi-session		Multi-session	12 wks	3x/wk	40 sessions	≤2 hrs	Physiological	BDNF (Brain-Derived Neurotrophic Factor)	10	473.39	5.3400	11	498.0700	19.3100	24.6800	14.3200	1.7200	1.7200	0.2409	52	52
9	Shin, M.J.	2016	RCT																IGF-1 (Insulin-like Growth Factor-1)	10	143.20	26.4200	11	178.1800	38.3800	34.9800	33.5900	1.0400	1.0400	0.1997	53	53
9	Shin, M.J.	2016	RCT																VEGF (Vascular Endothelial Growth Factor)	10	39.08	12.7700	11	35.1800	9.8300	-3.9000	11.4700	-0.3400	-0.3400	0.1785	54	54

Study No.	First Author	Year	Study Design	Participant Characteristics	Age Range	Setting	Intervention Type	Intervention Content	Control Program	Outcome Domain	Program Format	Residential Nights	Session Format	Total Duration (Weeks)	Sessions/ Week	Total Sessions	Session Duration	Outcome Category	Outcome Measure	Control n	Control Mean	Control SD	Exp n	Exp Mean	Exp SD	Mean Diff	Pooled SD	Hedges' g	yi	vi	effect_id	study_id
10	Oh, J.Y.	2015	NRCT	General Women	48.40±8.34	Forest Trail	Structured	Forest therapy for stress, fatigue, depression relief and vitality recovery	Forest therapy group, urban forest therapy group, non-therapy group	Psychological	Same-day/Multi-session		Multi-session	4 wks	1x/wk	10 sessions	≤6 hrs	Psychological	Stress	20	-62.80	22.2600	20	-39.4000	5.8000	23.4000	17.7900	1.3200	1.3200	0.1170	55	55
10	Oh, J.Y.	2015	NRCT																Fatigue	20	-81.15	38	20	-60.8500	13.6800	20.3000	30.0700	0.6800	0.6800	0.1016	56	56
10	Oh, J.Y.	2015	NRCT																Depression	20	-79.95	21.2500	20	-49.0500	3.7000	30.9000	16.8600	1.8300	1.8300	0.1363	57	57
11	Yoo, S.R.	2015	NRCT	General Women	54–64 yrs	Forest Trail	Unstructured	Forest walking	Flat ground walking group / Forest walking group	Physical	Same-day/Multi-session		Multi-session	10 wks	3x/wk	30 sessions	≤2 hrs	Physical	Cardiorespiratory Endurance	7	28.82	3.2000	7	29.0400	2.3000	0.2200	2.7800	0.0800	0.0800	0.2506	58	58
11	Yoo, S.R.	2015	NRCT																Right Extensor Strength	7	144.67	46.1300	7	177.7700	37.7400	33.1000	41.7100	0.7900	0.7900	0.2699	59	59
11	Yoo, S.R.	2015	NRCT																Right Extensor Strength	7	636.38	120.0900	7	667.5800	143.1800	31.2000	132.4200	0.2400	0.2400	0.2522	60	60
11	Yoo, S.R.	2015	NRCT																Flexibility	7	16.29	7.5600	7	19.4300	6.5200	3.1400	7.0100	0.4500	0.4500	0.2567	61	61
11	Yoo, S.R.	2015	NRCT																Body Fat Percentage	7	-18.34	4.7200	7	-15.7000	4.9500	2.6400	4.8300	0.5500	0.5500	0.2599	62	62
11	Yoo, S.R.	2015	NRCT																IN LINE-LUNGE	7	2.86	0.3780	7	2.1400	0.3780	-0.7200	0.3800	-1.9000	-1.9000	0.3634	63	63
11	Yoo, S.R.	2015	NRCT																PUSH-UP	7	1.57	0.5350	7	1.7100	0.3780	0.1400	0.4500	0.3100	0.3100	0.2534	64	64
11	Yoo, S.R.	2015	NRCT																Rotary Stability	7	2.29	0.4880	7	2.1400	0.3780	-0.1500	0.4400	-0.3400	-0.3400	0.2540	65	65
12	Yoon, M.A.	2020	NRCT	General Women	51–58 yrs	Forest Trail	Unstructured	Forest walking	Forest exercise group / No-exercise group	Psychological	Same-day/Multi-session		Multi-session	12 wks	1x/wk	20 sessions	≤2 hrs	Psychological	Stress Index	10	-49.1	2.9200	10	-36	3.0100	13.1000	2.9600	4.4300	4.4300	0.6335	66	66
13	Lee, M.O.	2018	NRCT	General Women	51–57 yrs	Forest Trail	Unstructured	Forest walking	Forest walking / Track walking	Physiological	Same-day/Multi-session		Multi-session	12 wks	3x/wk	20 sessions	≤2 hrs	Physiological	T-Cell	10	71.72	6.7100	10	73	6.8700	1.2800	6.7900	0.1900	0.1900	0.1843	67	67
13	Lee, M.O.	2018	NRCT																B-Cell	10	14.12	4.3200	10	16.2300	2.9900	2.1100	3.6700	0.5800	0.5800	0.1912	68	68
13	Lee, M.O.	2018	NRCT																NK Cell	10	21.43	5.3500	10	24.8700	3.0800	3.4400	4.2800	0.8000	0.8000	0.1981	69	69
13	Lee, M.O.	2018	NRCT																T-Cell	10	70.75	7.8000	10	71.4700	7.2600	0.7200	7.5300	0.1000	0.1000	0.1837	70	70
13	Lee, M.O.	2018	NRCT																B-Cell	10	13.43	2.9700	10	14.0600	3.4000	0.6300	3.2000	0.2000	0.2000	0.1844	71	71
13	Lee, M.O.	2018	NRCT																NK Cell	10	31.02	7.8300	10	35.7800	6.7600	4.7600	7.2800	0.6500	0.6500	0.1931	72	72
13	Lee, M.O.	2018	NRCT																Melatonin	10	126.32	10.5500	10	145.1700	131.3500	18.8500	96.3400	0.2000	0.2000	0.1844	73	73
13	Lee, M.O.	2018	NRCT																Melatonin	10	176.76	33.8600	10	219.9000	135.4600	43.1400	103.3800	0.4200	0.4200	0.1875	74	74
14	Lee, Y.J.	2020	RCT	Postmenopausal Women	53.4±2.68 yrs	Indoor	Indirect	Aroma necklace (essential oil inhalation)	Inhalation group / Control group	Psychological	Same-day/Multi-session		Multi-session	8 wks	1x/wk	10 sessions	Not specified	Psychological	Tension	21	-2.30	0.6800	20	-1.8800	0.7100	0.4200	0.6900	0.6100	0.6100	0.0982	75	75
14	Lee, Y.J.	2020	RCT																Anger	21	-2.10	0.7800	20	-1.8300	0.4200	0.2700	0.6300	0.4300	0.4300	0.0960	76	76
14	Lee, Y.J.	2020	RCT																Depression	21	-2.14	0.9100	20	-1.5800	0.5100	0.5600	0.7600	0.7400	0.7400	0.1003	77	77
14	Lee, Y.J.	2020	RCT																Vitality	21	3.00	0.4600	20	3.5100	0.6700	0.5100	0.5900	0.8700	0.8700	0.1028	78	78
14	Lee, Y.J.	2020	RCT																Fatigue	21	-2.84	0.9900	20	-2.3100	0.9000	0.5300	0.9500	0.5600	0.5600	0.0976	79	79
14	Lee, Y.J.	2020	RCT																Confusion	21	-2.32	0.8700	20	-2.3600	0.6400	-0.0400	0.7600	-0.0500	-0.0500	0.0939	80	80
14	Lee, Y.J.	2020	RCT																Sense of Competence	21	4.28	0.5600	20	5.6100	0.3800	1.3300	0.4600	2.8700	2.8700	0.1905	81	81
14	Lee, Y.J.	2020	RCT																Mental Well-being	21	4.43	0.6700	20	5.5400	0.3500	1.1100	0.5100	2.1600	2.1600	0.1486	82	82
14	Lee, Y.J.	2020	RCT																Stability	21	4.59	0.7500	20	6.0600	0.5200	1.4700	0.6300	2.3500	2.3500	0.1586	83	83
14	Lee, Y.J.	2020	RCT																Physical Well-being	21	4.92	0.7200	20	5.4500	0.3600	0.5300	0.5600	0.9500	0.9500	0.1045	84	84
14	Lee, Y.J.	2020	RCT																Vitality	21	4.60	0.5400	20	6.4500	0.7600	1.8500	0.6800	2.7200	2.7200	0.1806	85	85
15	Lee, J.S.	2016	RCT	General Women	59.32±7.11	Forest Bathing Site	Unstructured	Forest walking	Forest exercise group / Flat ground exercise group	Physical/Psychological	Same-day/Multi-session		Multi-session	12 wks	3x/wk	40 sessions	≤2 hrs	Physical	Upper Body Strength	9	35.43	5.0700	11	34.1000	4.1200	-1.3300	4.6300	-0.2900	-0.2900	0.1872	86	86
15	Lee, J.S.	2016	RCT																Lower Body Strength	9	23.20	6.6600	11	26	6.5500	2.8000	6.6000	0.4200	0.4200	0.1894	87	87
15	Lee, J.S.	2016	RCT																Upper Body Flexibility	9	1.15	5.7000	11	3.9600	6.1700	2.8100	6.1500	0.4600	0.4600	0.1902	88	88
15	Lee, J.S.	2016	RCT																Lower Body Flexibility	9	18.35	7.0700	11	21.3000	5.6900	2.9500	6.3600	0.4600	0.4600	0.1902	89	89
15	Lee, J.S.	2016	RCT																Agility-Balance	9	4.75	0.2900	11	4.3100	0.6300	-0.4400	0.4800	-0.9200	-0.9200	0.2047	90	90
15	Lee, J.S.	2016	RCT																Cardiorespiratory Endurance	9	636.78	32.5500	11	683.5000	41.9700	46.7200	37.7200	1.2400	1.2400	0.2206	91	91

Study No.	First Author	Year	Study Design	Participant Characteristics	Age Range	Setting	Intervention Type	Intervention Content	Control Program	Outcome Domain	Program Format	Residential Nights	Session Format	Total Duration (Weeks)	Sessions/ Week	Total Sessions	Session Duration	Outcome Category	Outcome Measure	Control n	Control Mean	Control SD	Exp n	Exp Mean	Exp SD	Mean Diff	Pooled SD	Hedges' g	yi	vi	effect_id	study_id
15	Lee, J.S.	2016	RCT																BMI	9	-23.01	7.4300	11	-22.9700	4.1100	0.0400	6.0100	0.0100	0.0100	0.1853	92	92
15	Lee, J.S.	2016	RCT															Psychological	Self-efficacy	9	80.38	8.7400	11	84.1400	6.7700	3.7600	7.7900	0.4800	0.4800	0.1906	93	93
15	Lee, J.S.	2016	RCT																Total Stress Score	9	-25.43	8.8800	11	-17.4300	7.0900	8	8.1900	0.9800	0.9800	0.2073	94	94
16	Jeong, S.R.	2019	NRCT	General Women	50–60 yrs	Forest Bathing Site	Structured	One-day intensive program	Experimental group / Control group	Psychological	Same-day /Multi-session		Multi-session	6 wks	2x/wk	20 sessions	≤2 hrs	Psychological	Ego Identity	11	3.08	0.1640	11	3.8300	0.1640	0.7500	0.1600	4.5700	4.5700	0.6076	95	95
16	Jeong, S.R.	2019	NRCT																Resilience	11	2.97	0.1660	11	3.9800	0.2950	1.0100	0.2500	4.0100	4.0100	0.5065	96	96
16	Jeong, S.R.	2019	NRCT																Psychological Well-being	11	3.07	0.1310	11	3.8900	0.3810	0.8200	0.3000	2.7100	2.7100	0.3227	97	97
16	Jeong, S.R.	2019	NRCT																Climacteric Symptoms	11	-34.55	1.5080	11	-24	2.7570	10.5500	2.1200	4.9900	4.9900	0.6920	98	98
17	Jeong, Y.G.	2023	RCT	General Women	40s–50s	Forest Trail	Unstructured	Integrated program using five senses and mental resilience	20 randomly assigned per group (experimental/control); pre-post test design	Psychological/Physiological	Same-day /Multi-session		Multi-session	2 wks	1x/wk	10 sessions	≤6 hrs	Psychological	Perceived Stress (PSS)	20	-2.885	0.1720	20	-2.0270	0.2160	0.8600	0.1900	4.4600	4.4600	0.3349	99	99
17	Jeong, Y.G.	2023	RCT																Leisure Satisfaction (LSS)	20	2.381	0.1500	20	3.2270	0.4300	0.8500	0.3500	2.4100	2.4100	0.1658	100	100
17	Jeong, Y.G.	2023	RCT															Physiological	Ubio Pulse Wave Test	20	-38.040	12.9460	20	-35.4050	13.4780	2.6400	13.2100	0.2000	0.2000	0.0965	101	101
18	Jeong, H.J.	2023	NRCT	General Women	40–64 yrs	Forest Trail	Unstructured	Forest barefoot walking	Barefoot group / Shod group	Psychological	Same-day /Multi-session		Multi-session	4 wks	2x/wk	10 sessions	≤2 hrs	Psychological	Perceived Stress (PSS)	9	-14.78	3.4900	9	-13.3300	4.1200	1.4500	3.8000	0.3800	0.3800	0.2052	102	102
18	Jeong, H.J.	2023	NRCT																Physiological Stress Index	9	-45.11	13.0700	9	-46.3300	15.7600	-1.2200	14.5000	-0.0800	-0.0800	0.2017	103	103
18	Jeong, H.J.	2023	NRCT																Vascular Health Index	9	1.78	8.5400	9	3.1100	16.2400	1.3300	14.5700	0.0900	0.0900	0.2018	104	104
19	Choi, G.	2023	NRCT	General Women	40–65 yrs	Forest Trail	Structured	Forest walking, loving-kindness meditation, breathing meditation	No forest therapy activities (control)	Psychological	Same-day /Multi-session		Multi-session	10 wks	1x/wk	10 sessions	≤2 hrs	Psychological	Depression	11	-1.311	0.2750	11	-1.2030	0.1750	0.1100	0.2300	0.4700	0.4700	0.1729	105	105
19	Choi, G.	2023	NRCT																Self-esteem	11	3.136	0.5530	11	3.3810	0.1070	0.2500	0.3900	0.6300	0.6300	0.1766	106	106
19	Choi, G.	2023	NRCT																Stress	11	-1.848	0.3840	11	-1.5380	0.3600	0.3100	0.3700	0.8300	0.8300	0.1828	107	107
20	Choi, J.H.	2010	NRCT	General Women	45–54 yrs	Forest Trail	Unstructured	Forest walking	Forest exercise group / Indoor exercise group	Physiological	Same-day /Multi-session		Multi-session	10 wks	3x/wk	30 sessions	≤2 hrs	Physiological	HDL-C	10	52.700	13.5980	10	50.4000	9.7320	-2.3000	11.8700	-0.1900	-0.1900	0.1843	108	108
20	Choi, J.H.	2010	NRCT																LDL-C	10	-117.8	22.5480	10	-115.4000	35.3720	2.4000	29.6000	0.0800	0.0800	0.1836	109	109
20	Choi, J.H.	2010	NRCT																TG (Total Cholesterol)	10	-136.9	57.2970	10	-121.8000	50.4070	15.1000	54.1600	0.2800	0.2800	0.1853	110	110
21	Choi, J.H.	2018	RCT	General Women	56.57±4.	Forest Trail	Unstructured	Forest walking	Forest walking exercise group / Indoor exercise group	Physical	Same-day /Multi-session		Multi-session	10 wks	3x/wk	30 sessions	≤2 hrs	Physical	Cardiorespiratory Endurance	9	35.43	5.0700	11	34.1000	4.1200	-1.3300	4.6300	-0.2900	-0.2900	0.1872	111	111
21	Choi, J.H.	2018	RCT																Muscle Strength (Extensor)	9	23.20	6.6600	11	26	6.5500	2.8000	6.6000	0.4200	0.4200	0.1894	112	112
21	Choi, J.H.	2018	RCT																Muscle Strength (Flexor)	9	1.15	5.7000	11	3.9600	6.1700	2.8100	6.1500	0.4600	0.4600	0.1902	113	113
21	Choi, J.H.	2018	RCT																Muscle Endurance (Extensor)	9	18.35	7.0700	11	21.3000	5.6900	2.9500	6.3600	0.4600	0.4600	0.1902	114	114
21	Choi, J.H.	2018	RCT																Muscle Endurance (Flexor)	9	4.75	0.2900	11	4.3100	0.6300	-0.4400	0.4800	-0.9200	-0.9200	0.2047	115	115
21	Choi, J.H.	2018	RCT																Flexibility	9	636.78	32.5500	11	683.5000	41.9700	46.7200	37.7200	1.2400	1.2400	0.2206	116	116
21	Choi, J.H.	2018	RCT																Body Fat Mass	9	-23.01	7.4300	11	-22.9700	4.1100	0.0400	6.0100	0.0100	0.0100	0.1853	117	117
22	Choi, J.H.	2016	RCT	General Women	52.37±2.43 yrs	Forest Trail	Unstructured	Forest walking	Treadmill walking + dumbbell exercise (1 kg); Forest exercise group / Flat exercise group	Physiological	Same-day /Multi-session		Multi-session	10 wks	3x/wk	30 sessions	≤2 hrs	Physiological	HDL-C	12	51.74	10.2000	12	53.3700	7.7000	1.6300	9.0200	0.1800	0.1800	0.1560	118	118

Study No.	First Author	Year	Study Design	Participant Characteristics	Age Range	Setting	Intervention Type	Intervention Content	Control Program	Outcome Domain	Program Format	Residential Nights	Session Format	Total Duration (Weeks)	Sessions/ Week	Total Sessions	Session Duration	Outcome Category	Outcome Measure	Control n	Control Mean	Control SD	Exp n	Exp Mean	Exp SD	Mean Diff	Pooled SD	Hedges' g	yi	vi	effect_id	study_id
22	Choi, J.H.	2016	RCT																LDL-C	12	-117.53	22.8000	12	-115.3400	31.4000	2.1900	27.4000	0.0800	0.0800	0.1555	119	119
22	Choi, J.H.	2016	RCT																TG	12	-140.44	46.7000	12	-120.5200	45.7000	19.9200	46.2400	0.4300	0.4300	0.1590	120	120
22	Choi, J.H.	2016	RCT																SOD (Antioxidant Enzyme)	12	2.84	1.7000	12	2.9300	0.2000	0.0900	1.2000	0.0800	0.0800	0.1555	121	121
22	Choi, J.H.	2016	RCT																Melatonin	12	16.31	1.6200	12	23.2200	2.3300	6.9100	2.0700	3.3400	3.3400	0.3720	122	122
23	Choi, J.H.	2016	RCT	General Women	53.9±2.69	Forest Trail	Unstructured	Forest walking	Forest walking / Track walking	Psychological	Same-day /Multi-session		Multi-session	12 wks	3x/wk	40 sessions	≤2 hrs	Psychological	Self-efficacy	9	80.38	8.7400	11	84.1400	6.7700	3.7600	7.7900	0.4800	0.4800	0.1906	123	123
23	Choi, J.H.	2016	RCT																Stress	9	-25.43	8.8800	11	-17.4300	7.0900	8	8.1900	0.9800	0.9800	0.2073	124	124
24	Hong, J.S.	2019	RCT	General Women	40s–50s	Indoor	Indirect	Fir essential oil aroma massage	Fir EO inhalation / Non-inhalation	Psychological	Same-day /Multi-session		Multi-session	2 wks	2x/wk	10 sessions	≤2 hrs	Psychological	Physical Stress	10	-0.87	0.1500	10	-0.6600	0.3200	0.2100	0.2500	0.8600	0.8600	0.2004	125	125
24	Hong, J.S.	2019	RCT																Psychological Stress	10	-0.95	0.2200	10	-0.5600	0.3500	0.3900	0.2900	1.3700	1.3700	0.2265	126	126
24	Hong, J.S.	2019	RCT																Physical Stress	10	-1.81	0.2800	10	-1.8600	0.3800	-0.0500	0.3300	-0.1500	-0.1500	0.1840	127	127
24	Hong, J.S.	2019	RCT																Psychological Stress	10	-1.28	0.2300	10	-0.6300	0.3200	0.6500	0.2700	2.4000	2.4000	0.3155	128	128
